# Effects of respiratory muscle training on swimming performance, respiratory muscle function, and pulmonary function of competitive swimmers: a systematic review and meta-analysis

**DOI:** 10.3389/fphys.2026.1770925

**Published:** 2026-03-20

**Authors:** Yunji Chen, Qing Yi, Kun Meng, Kuan Tao, Shuang Qin, Yang Yue, Dapeng Bao, Guole Jiang

**Affiliations:** 1 Basic Education College, National University of Defense Technology, Changsha, China; 2 Division of Sports Science and Physical Education, Tsinghua University, Beijing, China; 3 School of Physical Education, Hunan First Normal University, Changsha, China; 4 School of Sports Engineering, Beijing Sport University, Beijing, China; 5 School of Acupuncture-moxibustion and Tuina Rehabilitation, Hunan University of Chinese Medicine, Changsha, China; 6 China Institute of Sport and Health Science, Beijing Sport University, Beijing, China; 7 Medical Examination Center, Peking University Third Hospital, Beijing, China

**Keywords:** competitive swimmers, meta-analysis, pulmonary function, respiratory muscle training, swimming performance

## Abstract

**Background:**

Competitive swimming imposes unique physiological demands on the respiratory system. While respiratory muscle training (RMT) is proposed to optimize respiratory function and athletic performance, its efficacy in competitive swimmers remains inconsistent.

**Objectives:**

This systematic review and meta-analysis evaluated the effects of RMT on respiratory muscle function, pulmonary function, and swimming performance in competitive swimmers.

**Methods:**

Eight databases were searched from inception to 26 September 2025. Inclusion criteria were: (1) RMT interventions (inspiratory, expiratory, or combined) lasting ≥4 weeks; (2) participants were elite or high-level competitive swimmers; (3) control groups received sham RMT or standard training; and (4) outcomes included swimming performance, respiratory muscle strength, or pulmonary function. Methodological quality was assessed using Cochrane RoB 2.0 and the GRADE framework.

**Results:**

A total of 14 studies involving 375 competitive swimmers were included. The meta-analysis indicated that RMT significantly improved maximal inspiratory pressure (MIP) (SMD = 0.65, 95% CI: 0.29 to 1.01, *p* = 0.001) and certain dynamic pulmonary function parameters (forced expiratory volume in 1 s [FEV1], SMD = 0.45, 95% CI: 0.19 to 0.72, *p* = 0.001; forced inspiratory volume in the 1st second [FIV1], SMD = 0.41, 95% CI: 0.04 to 0.79, *p* = 0.03), as well as aerobic swimming performance (SMD = 1.02, 95% CI: 0.03 to 2.00, *p* = 0.04). In contrast, RMT did not significantly improve maximal expiratory pressure (MEP) (SMD = 0.16, 95% CI: −0.14 to 0.46, *p* = 0.30), other pulmonary function outcomes (forced vital capacity [FVC], maximal voluntary ventilation [MVV]), or sprint and middle-distance swimming performance (50 m, 100 m, and 200 m freestyle times, *p* > 0.05).

**Conclusion:**

RMT effectively enhances respiratory muscle strength and dynamic lung function in competitive swimmers across diverse levels, particularly with combined RMT demonstrating superior efficacy over IMT alone. Regarding athletic outcomes, RMT shows limited efficacy in enhancing short-distance swimming performance, while it exerts a significant positive impact on aerobic performance; however, its potential benefits require cautious interpretation due to high inter-study heterogeneity. This study offers updated insights into RMT’s role in aquatic conditioning, emphasizing the need for future load-matched trials to isolate its independent effects.

**Systematic Review Registration:**

https://www.crd.york.ac.uk/prospero/display_record.php?ID=CRD42024503624, identifier CRD42024503624.

## 1 Introduction

Swimming performance depends substantially on respiratory function (Cordain and Stager, 1988). The aquatic environment presents two distinct physiological challenges to competitive swimmers. The first challenge arises from hydrostatic pressure, which compresses the thoracic cavity and forces the respiratory muscles to work against increased resistance to maintain ventilation. This elevated work of breathing raises its metabolic cost and the risk of respiratory muscle fatigue, thereby directly limiting performance (Illidi et al., 2021; Yilmaz and Özdal, 2019). A second major constraint is imposed by the biomechanics of the stroke, which strictly limits breathing rhythm and demands highly efficient gas exchange within brief periods. Inadequate ventilation can lead to carbon dioxide retention and hypercapnia, subsequently accelerating muscle fatigue (Lavin et al., 2015; Wells et al., 2005). Therefore, sufficient respiratory muscle strength and pulmonary function are essential for competitive swimmers to sustain high-level performance.

Respiratory muscle training (RMT), including inspiratory muscle training (IMT), expiratory muscle training (EMT), combined, or breath-holding protocols, aims to improve respiratory endurance and/or strength through resistance-based or endurance-based strategies (HajGhanbari et al., 2013). Existing research indicated that RMT not only improves the functional capacity of respiratory muscles and reduces respiratory muscle fatigue (HajGhanbari et al., 2013) but may also enhance diaphragmatic and abdominal stability, thereby improving gliding efficiency and streamline posture, reducing water resistance, and enhancing athletic performance (Yoshida et al., 2020). Over the past decades, the role of RMT in enhancing athletic performance has been extensively investigated (Alnuman and Alshamasneh, 2022; Freitas et al., 2024; Tosun et al., 2025; Tsvetkova-Gaberska et al., 2023; Wu et al., 2021). For instance, in land-based sports such as cycling, running, and combat sports, several studies have demonstrated that RMT significantly improves respiratory function, exercise performance, and delays the onset of fatigue in athletes (Alnuman and Alshamasneh, 2022; Freitas et al., 2024; Tosun et al., 2025; Tsvetkova-Gaberska et al., 2023; Wu et al., 2021). However, studies investigating the effects of RMT on respiratory function and athletic performance in swimmers have yielded inconsistent results. For instance, Kilding et al. (2010), reported that a 6-week RMT program with a frequency of 14 sessions per week, significantly improved maximal inspiratory pressure and performance in the 100 m and 200 m events among competitive club-level swimmers. In contrast (Cunha et al., 2019), conducted a 12-week RMT program with a frequency of 5 sessions per week in elite swimmers but found no significant improvements in respiratory muscle strength, pulmonary function, or 200 m performance in the experimental group. These contrasting findings may be attributed to factors such as generally small sample sizes, heterogeneity in intervention protocols (e.g., training program, intensity, duration), and varying levels of swimmer expertise across studies. Given the heterogeneity of existing findings, there remains no clear consensus regarding the efficacy of RMT for competitive swimmers. A systematic review and meta-analysis are therefore warranted to establish its efficacy and to generate high-level evidence to guide clinical practice.

To date, while several systematic reviews have begun to address the application of RMT in swimming, yet critical gaps persist. For instance, Carvajal-Tello et al. (2024) examined pulmonary function in elite versus non-elite swimmers but omitted a quantitative synthesis of performance outcomes. Similarly, Illi et al. (2012) conducted a broad meta-analysis across multiple sports, which limited the depth of swimmer-specific insights. A more recent study by Liu et al. (2025) has evaluated swimming performance but overlooked the nuances of competitive tiers and event distances. Critically, these reviews often conflate recreational and elite populations, potentially masking the distinct physiological responses of high-level athletes. In this elite cohort, where the metabolic cost of breathing against hydrostatic pressure is intense, even marginal gains in pulmonary efficiency may yield significantly greater returns than in less-trained populations (Lavin et al., 2015; Yilmaz and Özdal, 2019). Consequently, there is a compelling need to scrutinize RMT-induced adaptations through the prism of elite physiology, where the interplay between respiratory mechanics and propulsive efficiency is most critical. This study seeks to move beyond broad outcome measures. We investigate whether RMT optimizes key physiological determinants, such as respiratory muscle strength and dynamic ventilatory capacity. The goal is to see how these improvements translate into functional advantages in the water. To this end, we implemented stringent inclusion criteria to evaluate not only respiratory muscle strength but also a comprehensive suite of pulmonary parameters. Findings are stratified by competitive level and event distance to provide high-level, sport-specific evidence for clinical practice and future trial design.

## 2 Materials and methods

This systematic review and meta-analysis was conducted in accordance with the Preferred Reporting Items for Systematic Reviews and Meta-Analyses (PRISMA) 2020 statement (Page et al., 2021) and the Cochrane Handbook for Systematic Reviews of Interventions. The review protocol was registered prospectively with the International Prospective Register of Systematic Reviews (PROSPERO: CRD42024503624).

### 2.1 Literature search

A comprehensive and systematic literature search was conducted independently by two researchers (YC and KM) from database inception to 26 September 2025. The search strategy was structured around three core components: (1) intervention (e.g., respiratory muscle training, RMT, IMT, EMT); (2) population (e.g., competitive swimmers, aquatic athletes); and (3) outcomes (e.g., swimming performance, pulmonary function, respiratory muscle strength). Eight electronic databases were searched using Boolean logic (“AND”, “OR”) (Page et al., 2021): Web of Science, PubMed (including MEDLINE and PubMed Central), SPORTDiscus, ScienceDirect, Scopus, Cochrane Library, Embase, and ProQuest. Notably, the search strategy was expanded beyond the scope initially registered in PROSPERO to broaden the retrieval of relevant literature. To ensure a comprehensive evidence synthesis, the initial search strategy was refined and expanded (Supplementary Table S1); these adjustments have been reflected in the updated PROSPERO registration. The search was conducted without restrictions on language or publication type to maximize the retrieval of eligible studies. Additionally, supplementary sources such as Google Scholar and ResearchGate were consulted, and the reference lists of relevant articles were manually screened to identify further eligible studies.

### 2.2 Eligibility criteria

The inclusion and exclusion criteria were established in accordance with the PICOS (Population, Intervention, Comparator, Outcomes, Study Design) framework to ensure a systematic and transparent selection process. Population (P): Competitive swimmers classified as “Highly-Trained/National Level” or “Elite/International Level” according to the International Classification of Training and Performance Caliber (McKay et al., 2022). This includes athletes regularly competing at national or international levels, as well as club/university swimmers engaged in systematic, high-volume training. Studies involving recreational swimmers, developmental-level athletes, or non-swimmers were excluded. Intervention (I): The intervention consisted of RMT (specifically IMT, EMT, or a combination of both) with a duration of ≥4 weeks. Comparator (C): Control conditions included sham or placebo RMT interventions, or participants maintaining their standard training regimen without any form of RMT. Outcomes (O): Primary outcomes were respiratory muscle strength, specifically measured by maximal inspiratory pressure (MIP) and maximal expiratory pressure (MEP), and swimming performance, with a focus on 100 m and 200 m swim times. Secondary outcomes included pulmonary function parameters (FEV1, FIV1, FVC, and MVV) and aerobic performance. Following the methodological framework for RMT established by Illi et al. (2012), aerobic performance was further categorized into incremental exercise tests, constant load tests, and time trials. Furthermore, other standardized metrics reflecting aerobic capacity, such as FINA points (used to normalize performance across various swimming events), were incorporated to ensure a comprehensive assessment of endurance capacity. Study Design (S): Randomized controlled trials (RCTs) published in English were included.

Studies were excluded if they met any of the following criteria: (1) available only as abstracts, protocols, or with inaccessible or incomplete data; (2) published in languages other than English; (3) insufficient data for effect size calculation; (4) non-original research (e.g., reviews, commentaries, conference proceedings); (5) duplicate publications; (6) studies without a parallel control group (e.g., single-arm pre-post design).

### 2.3 Selection process

The retrieved records were imported and managed using EndNote 20 software. After removal of duplicates, two independent reviewers (YC and KM) screened the titles and abstracts of the remaining studies against the predetermined inclusion and exclusion criteria. Literature that clearly did not meet the criteria was excluded. The full texts of potentially eligible studies were then obtained and assessed in detail for final inclusion. The two reviewers subsequently cross-verified their selections through discussion. Any disagreements regarding study eligibility were resolved through discussion or, if necessary, by consulting a third reviewer (DB).

### 2.4 Data collection process

The data extraction process followed the guidelines of the Cochrane Handbook for Systematic Reviews of Interventions (Higgins and Green, 2008). Two review authors (YC and QY) independently extracted data using a standardized form, which included: first author, publication year, sample size, participant characteristics, RMT protocol, exercise intervention design, baseline and post-intervention values for all outcomes, and outcome measures. The mean change from baseline and its standard deviation (SD) for each outcome were then derived from these data. The primary outcomes were respiratory muscle strength (MIP and MEP) and swimming performance (50 m, 100 m, and 200 m freestyle times), as they were the most frequently reported and representative indicators. Secondary outcomes included pulmonary function parameters (FEV1, FIV1, FVC, MVV) and other performance-related measures. For data presented exclusively in figures, WebPlotDigitizer (Version 4.2) was used for extraction. Corresponding authors were contacted via email by the first author to request any missing critical data. Disagreements between the two extracting authors were resolved through consensus, with arbitration by two other authors (GJ and DB) if needed.

### 2.5 Quality assessment

In accordance with the PROSPERO registration protocol, the risk of bias of included randomized controlled trials was assessed using the Cochrane Risk of Bias 2.0 (RoB 2) tool (Sterne et al., 2019). Two independent reviewers conducted the assessments independently. This domain-based tool evaluates five specific bias domains: (1) bias arising from the randomization process; (2) bias due to deviations from intended interventions; (3) bias due to missing outcome data; (4) bias in measurement of the outcome; and (5) bias in selection of the reported result. Each domain was appraised through a series of signalling questions, leading to a domain-level judgement of “Low risk”, “Some concerns”, or “High risk”. Subsequently, an overall risk of bias judgement for each study was derived based on these domain-level judgements, following the RoB 2 algorithm: a study was rated as “Low risk” only if all domains were judged as low risk; “Some concerns” if at least one domain raised some concerns but none were high risk; and “High risk” if at least one domain was judged as high risk (Sterne et al., 2019). Any disagreements between the two reviewers (YC and KM) were resolved through discussion or by consultation with a third reviewer (DB or KT) until a consensus was reached.

The quality of evidence for outcomes was evaluated using the Grading of Recommendations Assessment, Development, and Evaluation (GRADE), which characterizes the evidence on the study limitations, imprecision, inconsistency, indirectness, and publication bias (Guyatt et al., 2011; Meader et al., 2014). Any score on which the two reviewers (YC and KM) disagreed was discussed with a third author (DB or KT) until a consensus was achieved.

### 2.6 Statistical analysis

All statistical analyses were conducted using RevMan 5.3 (Cochrane Collaboration, Oxford, United Kingdom) and Stata 16.0 (StataCorp LLC, College Station, TX, United States). Continuous outcomes were pooled using mean difference (MD) when the same measurement units were reported across studies, or standardized mean difference (SMD) when different units were used. Specifically, the MD was calculated as the difference in mean change from baseline to post-intervention between the intervention and control groups (Higgins and Thomas, 2019). The SMD was derived by dividing the MD by the pooled standard deviation. For studies reporting the MD with its standard error (SE), values from studies reporting mean ± standard deviation (SD) were converted accordingly to MD and SE for inclusion in the meta-analysis. The magnitude of the SMD was interpreted as follows: 0∼0.19 represents negligible effect, 0.2∼0.49 represents a small effect, 0.5∼0.79 represents moderate effect, and ≥0.8 represents large effect (Cohen, 1988). Statistical significance was set at *p* < 0.05.

The I^2^ statistic was interpreted as follows: 0∼40% might not be important; 30∼60% may represent moderate heterogeneity; 50∼90% may represent substantial heterogeneity; and 75∼100% may represent considerable heterogeneity. If heterogeneity was not significant (I^2^ <50%), the fixed effect model was adopted. If heterogeneity was significant (I^2^ ≥ 50%), a random-effects model was used (Bown and Sutton, 2010; Higgins and Thomas, 2019). Sensitivity analysis was performed to evaluate the robustness of the pooled results by sequentially removing one study at a time to determine if any single study significantly influenced the overall effect size (Higgins and Thomas, 2019). Publication bias was evaluated through visual inspection of funnel plots and formal testing using Egger’s regression test. Significant asymmetry (Egger’s regression test *p* < 0.05) prompted further sensitivity analysis using the Trim and Fill method (Duval and Tweedie, 2000).

Subgroup analyses were performed to identify potential moderators of RMT efficacy. Continuous programming variables were dichotomized using the median split method (Bairan et al., 2019), with the median determined when data from ≥3 studies were available (Illi et al., 2012). Stratified variables included intervention type (IMT vs. combined RMT), duration (≤6 vs. >6 weeks), frequency (<10 vs. ≥10 sessions/week), training intensity (Supplementary Table S3), and swimmer level (Trained vs. Highly Trained) (McKay et al., 2022). To ensure statistical rigor, comparisons were restricted to categories containing at least two studies.

## 3 Results

### 3.1 Search results

**FIGURE 1:**
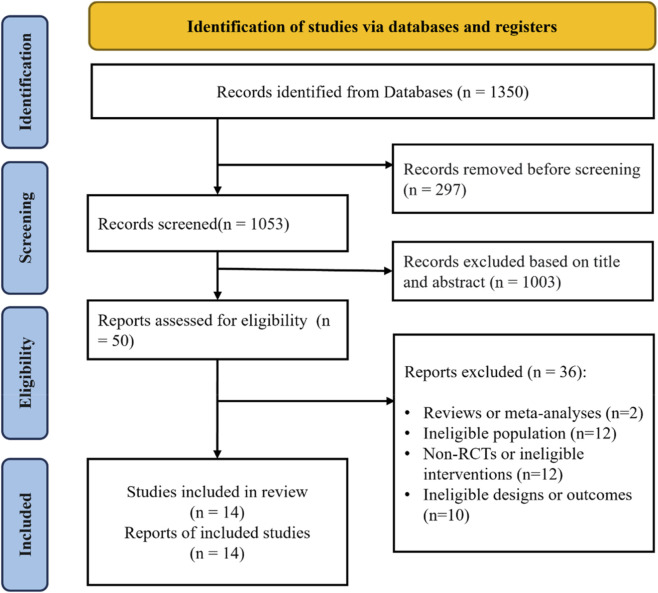
Study flowchart.

The systematic search of electronic databases yielded 1,350 potentially relevant records. After removing 297 duplicates, 1,053 records underwent title and abstract screening. Of these, 1,003 were excluded for not meeting the eligibility criteria. After initial screening of titles and abstracts, 50 full-text articles were retrieved and assessed for eligibility. During this stage, we ensured full-text access for all candidate records to avoid selection bias. Following a meticulous evaluation against the inclusion criteria, 36 studies were excluded for specific scientific reasons. Upon meticulous evaluation, 36 articles were excluded for the following specific reasons: (1) reviews or meta-analyses (n = 2), (2) ineligible population (n = 12), (3) non-randomized controlled trials (non-RCTs) or ineligible interventions (n = 12), and (4) ineligible study designs or outcomes (n = 10) (Patel et al., 2012). Ultimately, 14 studies (Ando et al., 2020; Cunha et al., 2019; Kapus, 2013; Kilding et al., 2010; Lemaitre et al., 2013; Lomax et al., 2019; Mickleborough et al., 2008; Ohya et al., 2022; Okrzymowska et al., 2019; Shei et al., 2016; Vašíčková et al., 2017; Wells et al., 2005; Wylegala et al., 2007; Yáñez-Sepúlveda et al., 2021) met all pre-defined inclusion criteria and were included in both the qualitative systematic review and the quantitative meta-analysis (Figure 1).

### 3.2 Characteristics of the studies included

Tables 1 and 2 summarize the characteristics of the 14 studies included in this systematic review and meta-analysis. Details regarding participant demographics, intervention protocols, comparators, and outcome measures are provided in the following sections.

**TABLE 1:** Characteristics of participants in each study.

Study	Total sample size	Age, year	Male	Female	EXP	CON	Athletic levels
Ohya et al. (2022) HIGH	20	20 ± 1	20	0	10	10	High level
Ohya et al. (2022) MOD	20	19 ± 1	20	0	10	10	High level
Yáñez-Sepúlveda et al. (2021)	15	15.1 ± 1.1	15	0	9	6	High level
Ando et al. (2020)	19	19.3 ± 0.1	19	0	10	9	Elite level
Cunha et al. (2019)	32	14–15	10	22	17	15	Elite level
Lomax et al. (2019) LOW	18	16 ± 3	11	7	9	9	High level
Lomax et al. (2019) HIGH	15	16 ± 1	7	8	8	7	High level
Okrzymowska et al. (2019)	16	18.3 ± 4.8	8	8	10	6	Elite level
Vašíčková et al. (2017)	20	EXP:12.0 ± 1.7 CON:11.5 ± 2.4	——	——	12	8	High level
Shei et al. (2016)	24	19.9 ± 2.6	12	12	8	8	High level
Kapus (2013)	12	14 ± 1	5	7	7	5	High level
Lemaitre et al. (2013)	20	16.5 ± 2.4	13	7	10	10	High level
Kilding et al. (2010)	16	19.1 ± 2.6	10	6	8	8	High level
Mickleborough et al. (2008)	20	18.2 ± 1.6	10	10	10	10	Elite level
Wylegala et al. (2007) RRMT	20	23.4 ± 4.3	20	0	10	10	High level
Wylegala et al. (2007) ERMT	20	23.4 ± 4.3	20	0	10	10	High level
Wells et al. (2005) 6 weeks—F	20	15.6 ± 1.3	0	20	10	10	High level
Wells et al. (2005) 12 weeks—F	14	15.6 ± 1.3	14	0	7	7	High level
Wells et al. (2005) 6 weeks—M	20	15.6 ± 1.3	0	20	10	10	High level
Wells et al. (2005) 12 weeks—M	14	15.6 ± 1.3	14	0	7	7	High level

EXP, experimental group; CON, control group; ——, no mentioned.

**TABLE 2:** Characteristics of respiratory muscle training.

Study (author, year)	Intervention					Comparator	Outcome measures		
	Intervention method	Type & device	Training intensity	Duration (week)	Frequency (session/week)		Respiratory muscle function	Pulmonary function	Swimming performance
Ohya et al. (2022)	IMT	POWERbreath IM trainer	75% MIP	6	12	continue their usual swimming	MIP↑	MVV↔	100-m freestyle swimming↓
Ohya et al. (2022)	IMT	POWERbreath IM trainer	50% MIP	6	12	continue their usual swimming	MIP↑*	MVV↔	100-m freestyle swimming↓
Yáñez-Sepúlveda et al. (2021)	IMT	POWERbreathe Classic Competition	50% MIP, increased by 5% weekly	4	7	Sham IMT (15% MIP)	MIP↑*	MVV↑ FVC↑	50-m freestyle swimming↓ 100-m freestyle swimming↓ 200-m freestyle swimming↓
Ando et al. (2020)	IMT	Inspiratory muscle training device (POWERbreathe Plus)	50% MIP	6	12	continue their usual swimming	MIP↑*	——	——
Cunha et al. (2019)	IMT	POWERbreathe Plus PB-2002	50% MIP	12	5	Sham IMT (15% MIP)	MIP↑	FEV1↔ FVC↑	FINA points↔
Lomax et al. (2019) LOW	IMT	Pressure-threshold IMT using POWERbreathe and Power Lung	50% MIP, increased periodically	6	14	Usual training only	MIP↑* MEP↔	FEV1↔ FVC↔	100-m freestyle swimming↔ 200-m freestyle swimming↔
Lomax et al. (2019)HIGH	IMT	Pressure-threshold IMT using POWERbreathe and Power Lung	50% MIP, increased periodically	6	14	Usual training only	MIP↑* MEP↔	FEV1↔ FVC↔	100-m freestyle swimming↔ 200-m freestyle swimming↔
Okrzymowska et al. (2019)	IMT	Threshold IMT (Respironics)	30–60% MIP	8	10	Standard training	MIP↑* MEP↑	FEV1↑ FVC↑	——
Vašíčková et al. (2017)	RMT (RMST & RMET)	Threshold IMT & Threshold PEP	30% of MIP and MEP, increased by 2 cm H_2_O weekly	4	7	Standard swimming training without RMT	MIP↑* MEP↔	FEV1↔ FVC↔	
Shei et al. (2016)	IMT	RT2 trainer	80% MIP, progressively increased work-rest ratios until task failure	12	3	Sham IMT	MIP↑*	FEV1↔ FIV1↑* FVC↔	——
Kapus (2013)	IMT	POWERbreathe	50% MIP, increased weekly	6	14	Sham IMT (∼15% MIP)	MIP↑* MEP↑	FEV1↑	50-m freestyle swimming↔ 100-m freestyle swimming↓*
Lemaitre et al. (2013)	RMT (RMET)	SpiroTiger (Idiag AG, Fehraltorf, Switzerland)	60% MVV, adjusted weekly	8	5	Standard training	MIP↑* MEP↑*	FEV1↔ FVC↑ MVV↑	50-m freestyle swimming↓* 200-m freestyle swimming↓*
Kilding et al. (2010)	IMT	Pressure threshold device (POWERbreathe)	50% MIP, increased weekly	6	14	Sham IMT (15% MIP)	MIP↑*	FEV1↔ FVC↔	100-m freestyle swimming↓* 200-m freestyle swimming↓*
Mickleborough et al. (2008)	IMT	Flow-resistive device (RT2 trainer)	80% SMIP	12	3	Sham IMT (30% MIP)	MIP↑ MEP↑	FIV1↑ FEV1↑ FVC↑	——
Wylegala et al. (2007)-RRMT	RMT (RMST)	Custom breathing apparatus with pressure threshold valves	50 cm H_2_O	4	5	Placebo RMT (breath-holds)	MIP↑* MEP↑*	FEV1↔ FVC↔ MVV↑*	Time to exhaustion↑*
Wylegala et al. (2007)-ERMT	RMT (EMET)	Endurance RMT (isocapnic hyperpnea) with custom breathing apparatus	60% MVV, adjusted weekly	4	5	Sham RMT (10% MIP/MEP)	MIP↔ MEP↔	FEV1↑* FVC↑* MVV↑*	Time to exhaustion↑*
Wells et al. (2005) 6 weeks—F	RMT (IMT & EMT)	PowerLung	50–60% MIP/MEP	69	10	Sham CRMT (10% MIP/MEP)	MIP↔ MEP↔	FEV1↑ FIV1↑ MVV↑	——
Wells et al. (2005) 12 weeks—F	RMT	PowerLung	70–80% MIP/MEP	12	10	Moderate RMT (50%–60% MIP/MEP)	MIP↑ MEP↔	FEV1↑ FIV1↑ MVV↑	——
Wells et al. (2005) 6 weeks—M	RMT	PowerLung	50–60% MIP/MEP	6	10	Sham CRMT (10% MIP/MEP)	MIP↔ MEP↔	FEV1↑ FIV1↑ MVV↑	——
Wells et al. (2005) 12 weeks—M	RMT	PowerLung	70–80% MIP/MEP	12	10	Moderate RMT (50%–60% MIP/MEP)	MIP↔ MEP↔	FEV1↑ FIV1↑ MVV↑	——

EXP, experimental group; CON, control group; MIP, maximal inspiratory pressure; MEP, maximal expiratory pressure; RMT, respiratory muscle training; IMT, inspiratory muscle training; EMT, expiratory muscle training; RMST, respiratory muscle strength training; RMET, respiratory muscle endurance training; FEV1, Forced expiratory volume in 1 s; FIV1, Forced Inspiratory Volume in the 1st second; FVC, forced vital capacity; MVV, maximal voluntary ventilation; ↑, value increased; ↑*, value significantly increased (*p* < 0.05); ↓, value decreased; ↓*, value significantly decreased (*p* < 0.05); ↔, no change. For swimming time trials, ↓ * denotes a statistically significant improvement in performance (reduction in time).

#### 3.2.1 Participant characteristics

A total of 14 studies involving competitive swimmers were included in this systematic review and meta-analysis. The total number of participants across all studies was 375, with ages ranging from 11 to 23 years. The majority of participants were male, though several studies included mixed or female-only cohorts (Cunha et al., 2019; Kapus, 2013; Kilding et al., 2010; Lemaitre et al., 2013; Shei et al., 2016; Wells et al., 2005). Participants were classified as either “Highly-Trained/National Level” or “Elite/International Level” according to the International Classification of Training and Performance Caliber (McKay et al., 2022). Among the included studies, four were conducted in elite/international level swimmers (Ando et al., 2020; Cunha et al., 2019; Mickleborough et al., 2008; Okrzymowska et al., 2019), while the remaining studies involved highly-trained/national level athletes. The EXP and CON groups were comparable in sample size across studies, with group sizes typically ranging from 6 to 17 participants.

#### 3.2.2 Intervention characteristics

##### 3.2.2.1 Type of intervention

The primary intervention was RMT, which included IMT (Ando et al., 2020; Cunha et al., 2019; Kapus, 2013; Kilding et al., 2010; Lomax et al., 2019; Mickleborough et al., 2008; Ohya et al., 2022; Okrzymowska et al., 2019; Shei et al., 2016; Vašíčková et al., 2017; Yáñez-Sepúlveda et al., 2021), EMT (Wylegala et al., 2007), or a combination of both (Lemaitre et al., 2013; Vašíčková et al., 2017; Wells et al., 2005; Wylegala et al., 2007). IMT was the predominant modality, implemented in 12 studies. Regarding training equipment, pressure-threshold loading devices were the most frequently used, including the POWERbreathe (Ando et al., 2020; Cunha et al., 2019; Kapus, 2013; Kilding et al., 2010; Lomax et al., 2019; Ohya et al., 2022; Yáñez-Sepúlveda et al., 2021), PowerLung (Lomax et al., 2019; Wells et al., 2005), and the RT2 trainer (Mickleborough et al., 2008; Shei et al., 2016), while one study utilized a custom-designed breathing apparatus (Wylegala et al., 2007).

##### 3.2.2.2 Training intensity

Training intensity varied across studies, typically set as a percentage of MIP or MEP. Common intensities ranged from 30% to 80% of MIP (Ando et al., 2020; Cunha et al., 2019; Kapus, 2013; Kilding et al., 2010; Lomax et al., 2019; Mickleborough et al., 2008; Ohya et al., 2022; Okrzymowska et al., 2019; Shei et al., 2016; Vašíčková et al., 2017; Wells et al., 2005; Yáñez-Sepúlveda et al., 2021), with some studies applying progressive overload by increasing intensity weekly (e.g., by 5% of MIP (Kapus, 2013; Kilding et al., 2010; Yáñez-Sepúlveda et al., 2021) or by 2 cmH_2_O (Vašíčková et al., 2017)). Two studies used isocapnic hyperpnea at 60% of MVV (Lemaitre et al., 2013; Wylegala et al., 2007).

##### 3.2.2.3 Duration

The intervention duration ranged from 4–12 weeks, with the majority of studies implementing programs lasting 6–8 weeks (Ando et al., 2020; Kapus, 2013; Kilding et al., 2010; Lemaitre et al., 2013; Lomax et al., 2019; Ohya et al., 2022; Okrzymowska et al., 2019; Shei et al., 2016; Vašíčková et al., 2017; Wylegala et al., 2007). Four studies applied longer interventions of 12 weeks (Cunha et al., 2019; Mickleborough et al., 2008; Shei et al., 2016; Wells et al., 2005).

##### 3.2.2.4 Frequency

Training frequency varied from 3–14 sessions per week, with several studies implementing twice-daily sessions (e.g., 14 sessions/week (Kapus, 2013; Kilding et al., 2010; Lomax et al., 2019)). The most common frequency was 5–7 sessions per week (Cunha et al., 2019; Lemaitre et al., 2013; Vašíčková et al., 2017; Wylegala et al., 2007; Yáñez-Sepúlveda et al., 2021).

#### 3.2.3 Comparator groups

Control groups were designed to account for potential placebo effects and to isolate the specific physiological adaptations induced by RMT. These control conditions included: (1) sham or placebo RMT interventions, typically conducted at a low intensity (e.g., 10–15% of MIP) (Cunha et al., 2019; Kapus, 2013; Kilding et al., 2010; Mickleborough et al., 2008; Shei et al., 2016; Wells et al., 2005; Wylegala et al., 2007; Yáñez-Sepúlveda et al., 2021); (2) standard swimming training regimens without any form of respiratory muscle training (Ando et al., 2020; Lemaitre et al., 2013; Lomax et al., 2019; Ohya et al., 2022; Okrzymowska et al., 2019; Vašíčková et al., 2017); and (3) alternative RMT such as breath-holding or low-intensity respiratory maneuvers (Wylegala et al., 2007).

#### 3.2.4 Outcome measurements

The outcome measures across the included studies were systematically categorized into three domains. Respiratory muscle function was evaluated in 14 studies, with MIP assessed in all 14 studies and MEP reported in 8 studies (Kapus, 2013; Lemaitre et al., 2013; Lomax et al., 2019; Mickleborough et al., 2008; Okrzymowska et al., 2019; Vašíčková et al., 2017; Wells et al., 2005; Wylegala et al., 2007). Pulmonary function parameters were investigated in 13 studies. Specifically, FEV1 was measured in 9 studies (Cunha et al., 2019; Kilding et al., 2010; Lemaitre et al., 2013; Lomax et al., 2019; Mickleborough et al., 2008; Okrzymowska et al., 2019; Shei et al., 2016; Vašíčková et al., 2017; Wells et al., 2005), while FIV1 was assessed in 3 studies (Ohya et al., 2022; Wells et al., 2005; Wylegala et al., 2007). Additionally, FVC was recorded in 10 studies (Cunha et al., 2019; Kilding et al., 2010; Lemaitre et al., 2013; Lomax et al., 2019; Mickleborough et al., 2008; Okrzymowska et al., 2019; Shei et al., 2016; Vašíčková et al., 2017; Wylegala et al., 2007; Yáñez-Sepúlveda et al., 2021), and MVV was evaluated in 4 studies (Ohya et al., 2022; Wells et al., 2005; Wylegala et al., 2007; Yáñez-Sepúlveda et al., 2021). Swimming performance was examined in 9 studies. Outcome measures included race times for the 50 m (Kapus, 2013; Lemaitre et al., 2013; Wells et al., 2005; Yáñez-Sepúlveda et al., 2021), 100 m (Kilding et al., 2010; Lomax et al., 2019; Ohya et al., 2022; Wells et al., 2005; Yáñez-Sepúlveda et al., 2021), and 200 m freestyle events (Kilding et al., 2010; Lemaitre et al., 2013; Lomax et al., 2019; Wells et al., 2005; Yáñez-Sepúlveda et al., 2021). Regarding aerobic performance, measurement protocols in the original studies varied to capture different physiological dimensions: Cunha et al. (2019) utilized FINA points—a standardized scoring system converted from time trials to allow for comparative analysis of elite performance—while Shei (2020) employed a time-to-exhaustion (TTE) protocol during sustained swimming to evaluate aerobic endurance capacity. All outcomes were measured at baseline and immediately post-intervention, with comparisons conducted both within and between groups.

### 3.3 Results of the meta-analysis

#### 3.3.1 Effect of RMT on respiratory muscle strength

##### 3.3.1.1 MIP

**FIGURE 2:**
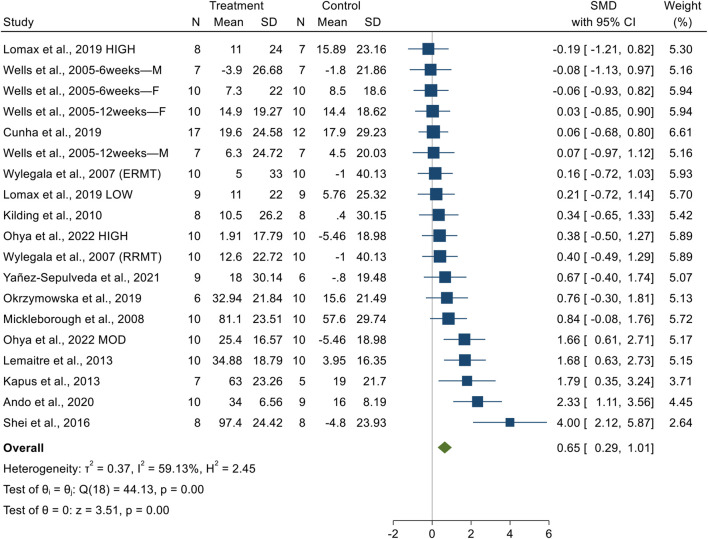
The forest plot of MIP.

Significant differences in MIP were observed between the experimental and control groups. Given the presence of moderate heterogeneity (I^2^ = 59.13%, *p* = 0.001), a random-effects model was adopted. The pooled SMD for post-intervention comparisons was 0.65 (95% CI: 0.29 to 1.01, *p* = 0.001), indicating a moderate effect size in favor of RMT (Figure 2). Although visual inspection of the funnel plot and Egger’s test (*t* = 5.08, *p* = 0.001) suggested potential publication bias (Supplementary Figures S2, S3), the Trim and Fill analysis imputed no missing studies (n = 0). Consequently, the adjusted effect size remained unchanged (SMD = 0.65), confirming the robustness of the primary results.

##### 3.3.1.2 MEP

**FIGURE 3:**
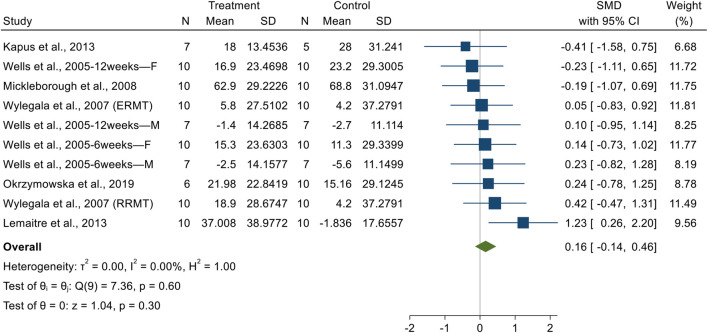
The forest plot of MEP.

No significant difference in MEP were observed between the experimental and control groups. Given the low heterogeneity (I^2^ = 0.00%, *p* = 0.60), a fixed-effect model was adopted. The pooled SMD for post-intervention comparisons was 0.16 (95% CI: −0.14 to 0.46, *p* = 0.30), indicating a trivial effect size in favor of RMT (Figure 3). Visual inspection of the funnel plot and Egger’s test (t = 0.001, *p* = 0.99) suggested no evidence of publication bias (Supplementary Figures S2, S3).

#### 3.3.2 Effect of RMT on pulmonary function

##### 3.3.2.1 FEV1

**FIGURE 4:**
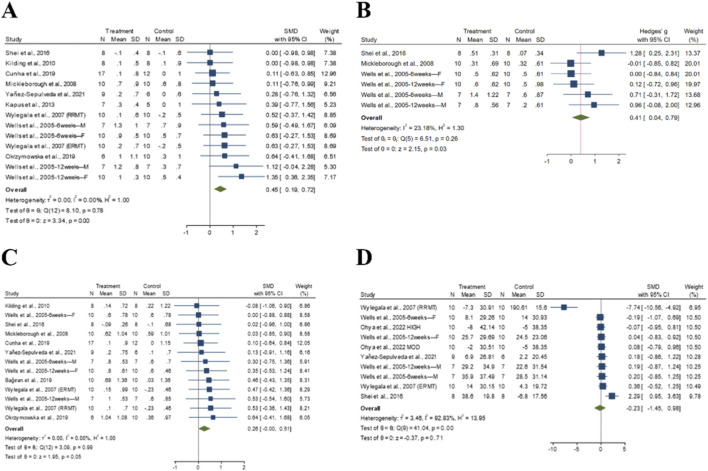
The forest plot of pulmonary function. **(A)** Forced Expiratory Volume in 1 Second. **(B)** Forced Expiratory Volume in the 1st second. **(C)** Forced Vital Capacity. **(D)** Maximal Voluntary Ventilation.

A significant difference in FEV1 were observed between the experimental and control groups. Given the low heterogeneity (I^2^ = 0.00%, *p* = 0.78), a fixed-effect model was adopted. The pooled SMD for post-intervention comparisons was 0.45 (95% CI: 0.19 to 0.72, *p* = 0.001), indicating a small effect size in favor of RMT (Figure 4A). The funnel plot and Egger’s test (t = 1.16, *p* = 0.27) suggested no evidence of publication bias (Supplementary Figures S2, S3).

##### 3.3.2.2 FIV1

A significant difference in FIV1 were observed between the experimental and control groups. Given the low heterogeneity (I^2^ = 23.18%, *p* = 0.26), a fixed-effect model was adopted. The pooled SMD for post-intervention comparisons was 0.41 (95% CI: 0.04 to 0.79, *p* = 0.03), indicating a small effect size in favor of RMT (Figure 4B). The funnel plot and Egger’s test (*t* = 2.43, *p* = 0.07) suggested no significant evidence of publication bias (Supplementary Figures S2, S3).

##### 3.3.2.3 FVC

No significant difference in FVC were observed between the experimental and control groups. Given the low heterogeneity (I^2^ = 0.001%, *p* = 0.99), a fixed-effect model was adopted. The pooled SMD for post-intervention comparisons was 0.26 (95% CI: −0.001 to 0.51, *p* = 0.05), indicating a small effect size in favor of RMT (Figure 4C). The funnel plot and Egger’s test (*t* = 0.46, *p* = 0.65) suggested no evidence of publication bias (Supplementary Figures S2, S3).

##### 3.3.2.4 MVV

No significant difference in MVV were observed between the experimental and control groups. Given the high heterogeneity (I^2^ = 92.83%, *p* = 0.001), a random-effects model was adopted. The pooled SMD for post-intervention comparisons was −0.23 (95% CI: −1.45 to 0.98, *p* = 0.71), indicating a small effect size in favor of RMT (Figure 4D). The funnel plot and Egger’s test (*t* = −3.67, *p* = 0.06) suggested no significant evidence of publication bias (Supplementary Figures S2, S3).

#### 3.3.3 Effect of RMT on swimming performance

##### 3.3.3.1 50 m freestyle time

**FIGURE 5:**
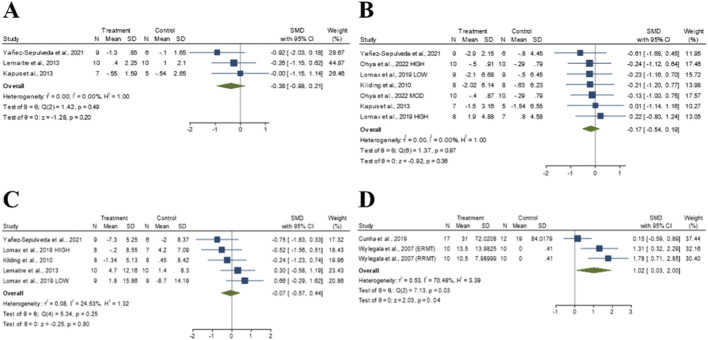
The forest plot of swimming performance. **(A)** 50 m Freestyle Time. **(B)** 100 m Freestyle Time. **(C)** 200 m Freestyle Time. **(D)** Aerobic Performance.

No significant difference in 50-m freestyle time were observed between the experimental and control groups. Given the low heterogeneity (I^2^ = 0.001%, p = 0.49), a fixed-effect model was adopted. The pooled SMD for post-intervention comparisons was −0.38 (95% CI: −0.98 to 0.21, p = 0.20), indicating a small effect size in favor of RMT (Figure 5A). Egger’s test (t = −0.15, *p* = 0.91) suggested no evidence of publication bias.

##### 3.3.3.2 100 m freestyle time

No significant difference in 100-m freestyle time were observed between the experimental and control groups. Given the low heterogeneity (I^2^ = 0.00%, *p* = 0.97), a fixed-effect model was adopted. The pooled SMD for post-intervention comparisons was −0.17 (95% CI: −0.54 to 0.19, *p* = 0.36), indicating a trivial effect size in favor of RMT (Figure 5B). Egger’s test (t = 0.08, *p* = 0.94) suggested no evidence of publication bias (Supplementary Figure S4).

##### 3.3.3.3 200 m freestyle time

No significant difference in 200-m freestyle time were observed between the experimental and control groups. Given the low heterogeneity (I^2^ = 24.53%, *p* = 0.25), a fixed-effect model was adopted. The pooled SMD for post-intervention comparisons was −0.07 (95% CI: −0.57 to 0.44, *p* = 0.80), indicating a trivial effect size in favor of RMT (Figure 5C). Egger’s test (*t* = −1.87, *p* = 0.16) suggested no evidence of publication bias (Supplementary Figure S4).

##### 3.3.3.4 Aerobic performance

A significant difference in aerobic performance were observed between the experimental and control groups. Given the substantial heterogeneity (I^2^ = 70.48%, *p* = 0.03), a random-effects model was adopted. The pooled SMD for post-intervention comparisons was 1.02 (95% CI: 0.03 to 2.00, *p* = 0.04), indicating a large effect size in favor of RMT (Figure 5D). Egger’s test (*t* = 2.67, *p* = 0.23) suggested no evidence of publication bias.

### 3.4 Subgroup analyses

The results of the subgroup analyses are summarized in Supplementary Table S4. Subgroup analysis revealed that the intervention type significantly modified only FEV1(P_*diff*_ = 0.03). Specifically, combined RMT yielded a large and significant improvement (SMD = 0.78; 95% CI: 0.38 to 1.17), whereas IMT alone did not reach statistical significance (SMD = 0.19; 95% CI: -0.17 to 0.55). In contrast, no significant between-group differences were observed for other respiratory outcomes (e.g., MIP, MEP, and FVC) when stratified by athletic levels, training duration, or frequency (all P_*diff*_ > 0.05). Lastly, inconsistent metrics and progressive loading protocols precluded a quantitative subgroup analysis of training intensity; therefore, these effects were synthesized qualitatively (Table 2).

### 3.5 Sensitivity analysis

Sensitivity analysis demonstrated the robustness of the findings. The pooled results for respiratory parameters (MIP, MEP, FEV1, FIV1, FVC, MVV) and swimming performance (100 m and 200 m freestyle times) remained consistent in both direction and magnitude, with no single study significantly influencing the overall effect sizes (Supplementary Figures S2, S3).

### 3.6 Risk of bias and GRADE

The methodological quality of the included studies was evaluated using the RoB 2 tool. As summarized in Supplementary Figure S1, the overall risk of bias across the 14 included studies was moderate. Most studies demonstrated a low risk of bias in the domains of outcome measurement and selection of the reported result; however, frequent concerns were identified in the randomization process and deviations from intended interventions. Specifically, three studies were classified as having a high overall risk of bias (Okrzymowska et al., 2019; Vašíčková et al., 2017; Yáñez-Sepúlveda et al., 2021). Nine studies raised some concerns regarding bias (Ando et al., 2020; Cunha et al., 2019; Kapus, 2013; Kilding et al., 2010; Lemaitre et al., 2013; Lomax et al., 2019; Mickleborough et al., 2008; Ohya et al., 2022; Wylegala et al., 2007). Only two studies were assessed as having a low overall risk of bias (Shei et al., 2016; Wells et al., 2005).

As shown in Supplementary Table S2, we evaluated the evidence syntheses using the GRADE approach. The certainty of the FEV1 results was rated as moderate due to potential risk of bias, whereas the FIV1 results were rated as moderate primarily due to imprecision (attributed to small sample size). The results for respiratory muscle strength (MIP, MEP) and other pulmonary functions (FVC, MVV) were assessed as having low to very low certainty, with the quality downgraded mainly because of a high risk of detection bias, significant statistical heterogeneity, and imprecision (wide confidence intervals) across the included studies.

## 4 Discussion

The findings of this systematic review and meta-analysis indicate that RMT significantly enhances inspiratory muscle strength (MIP) and specific dynamic ventilatory functions, including FEV1 and FIV1, in competitive swimmers. In contrast, current quantitative evidence does not support statistically significant improvements in expiratory muscle strength (MEP), pulmonary capacity (FVC), or maximal voluntary ventilation (MVV). Regarding swimming performance, RMT did not yield significant gains in sprint or middle-distance freestyle events (50–200 m). While preliminary evidence suggests potential benefits for aerobic performance, these findings warrant cautious interpretation due to the limited study count and substantial heterogeneity. Notably, subgroup analysis revealed a significant moderating effect of intervention type on FEV1, with RMT demonstrating superior efficacy compared to IMT alone.

### 4.1 Interpretation of results and comparison with previous research

#### 4.1.1 Effects of RMT on respiratory muscle function

The significant improvements in MIP identified in this meta-analysis offer preliminary evidence of enhanced inspiratory muscle function. These gains align with the established principles of muscle plasticity, whereby RMT-induced mechanical loading triggers structural adaptations—most notably fiber hypertrophy within the diaphragm and external intercostals—alongside enhanced neuromuscular efficiency (Ando et al., 2020; Kapus, 2013). Within the hydrodynamics of competitive swimming, such adaptations are of particular physiological significance; they are hypothesized to fortify the respiratory pump against the premature onset of fatigue, thereby potentially delaying the inspiratory muscle metaboreflex (Ando et al., 2020). Given that swimmers must already contend with the restrictive loads of hydrostatic pressure and horizontal positioning (Lemaitre et al., 2013), targeted RMT serves as a supramaximal stimulus that transcends the demands of routine aquatic training. This “overloading” effect further potentiates the functional reserve of the inspiratory musculature beyond what is attainable through swimming alone (Xavier et al., 2025). Consequently, our findings resonate with previous observations in elite aquatic athletes (Kilding et al., 2010; Lomax et al., 2019; Shei, 2020), reinforcing the role of RMT as a robust modality for enhancing respiratory strength.

#### 4.1.2 Effects of RMT on pulmonary function

Our analysis identifies a significant positive effect of RMT on dynamic ventilatory parameters, specifically FEV1 and FIV1. These findings indicate that RMT effectively enhances airflow velocity and the dynamic power of respiratory muscles, which are critical for overcoming the resistive loads of the aquatic environment (Carvajal-Tello et al., 2024). The improvement in FEV1 likely stems from enhanced forced expiratory power, a crucial adaptation for swimmers who must complete forceful exhalations within the brief breathing windows permitted by stroke biomechanics (Patel et al., 2012; Wells et al., 2005). These dynamic gains suggest a transition toward superior ventilatory efficiency rather than a fundamental expansion of static lung volumes, as FVC and MVV remained stable. This distinction aligns with previous investigations involving swimmers and divers (Kilding et al., 2010; Lomax et al., 2019; Shei, 2020). Specifically, it suggests that performance is primarily limited by the velocity of air exchange and the capacity for high-intensity ventilatory work, rather than static total lung capacity.

#### 4.1.3 Effect of RMT on swimming performance

Our quantitative analysis indicates that RMT did not elicit significant performance enhancements in 50-m, 100-m, and 200-m freestyle events. This lack of transfer typically aligns with the specific bioenergetics and mechanical demands of these distances. First, competitive swimming events ranging from 50 to 200 m demand intensities typically exceeding 85% of VO_2_max, relying predominantly on anaerobic metabolism (Wells et al., 2005). The ergogenic benefits of RMT—specifically, the delay of respiratory muscle fatigue and the subsequent attenuation of the respiratory muscle metaboreflex—are most pronounced in endurance events lasting longer than 2 minutes where aerobic metabolism dominates (Welch et al., 2019). Current evidence suggests that the metaboreflex (which triggers sympathetically mediated vasoconstriction in locomotor limbs) plays a negligible role during such short-duration, supramaximal efforts (Liu et al., 2025), thereby limiting the potential for RMT to improve performance in sprint and middle-distance swimming. Second, regarding training specificity, most RMT protocols prioritize pressure generation (strength) or sustained loading (endurance) rather than the velocity-specific adaptations required for sprinting. Sprint swimming necessitates explosive ventilatory maneuvers—specifically, rapid inhalation and exhalation during starts and turns. If RMT does not sufficiently enhance respiratory muscle power (force × velocity), it may fail to meet the demands for high-velocity airflow dynamics required during these phases (Wylegala et al., 2007).

Conversely, a statistically significant improvement was observed in aerobic performance measures. However, this finding must be interpreted with substantial caution. The high heterogeneity observed in this outcome likely stems from a complex interplay of contextual factors. Specifically, the baseline swimming training load may exert a “dose-dependent” effect on the efficacy of RMT, as previously reported by Mickleborough et al. (2008), where the marginal gains from RMT may be diminished in athletes already undergoing high-volume aquatic conditioning. Nevertheless, these preliminary observations are mechanistically consistent with the observed improvements in MIP and dynamic pulmonary function (FEV1, FIV1). Physiologically, stronger respiratory muscles operate at a lower relative intensity for a given ventilation, potentially reducing the oxygen cost of breathing (Kilding et al., 2010). For endurance swimming, this improved metabolic efficiency may spare oxygen for locomotor muscles, thereby enhancing swimming economy (Shei, 2018). As proposed by Wylegala et al. (2007), RMT may enhance the oxidative capacity of respiratory muscles, delay metabolite accumulation, and consequently attenuate the metaboreflex. This preservation of limb blood flow would theoretically sustain aerobic power output and improve endurance performance, although this hypothesis requires verification through larger-scale trials.

#### 4.1.4 Subgroup analyses

Subgroup analyses identified several key factors modulating RMT’s efficacy in competitive swimmers. Regarding intervention type, a significant moderating effect was found for FEV1, where combined RMT (IMT + EMT) outperformed IMT alone. This suggests that concurrent stimulation of both inspiratory and expiratory muscles provides a more comprehensive stimulus by more closely replicating the complete respiratory cycle and the high intrathoracic pressures of intense swimming (Tan et al., 2023). The benefits of RMT remained consistent across training variables and competitive level (p > 0.05). Positive trends in MIP and FEV1 were observed regardless of duration (≤6 vs. >6 weeks, frequency (<10 vs. ≥10 sessions/week), or level (elite vs. highly trained). This indicates that the respiratory system of swimmers remains responsive to RMT even at the highest performance tiers or during shorter interventions. The lack of significant differences may stem from the limited number of studies in certain subgroups, which could mask subtle physiological variations. While these results demonstrate RMT’s versatility, they also highlight the need for future load-matched trials to clarify optimal dosage and potential ceiling effects in elite cohorts.

The synthesis of sex-related differences remains a critical consideration in interpreting RMT efficacy. Due to a pervasive lack of sex-disaggregated data in the primary literature, a formal quantitative meta-analysis of between-sex differences could not be performed. Nevertheless, theoretical considerations suggest that female swimmers may exhibit distinct adaptive responses due to sex-specific respiratory mechanics, such as smaller airway diameters and a higher work of breathing at similar relative intensities compared to males (Mickleborough et al., 2008). Crucially, most included studies were predominantly centered on male cohorts, leaving a notable evidence gap for elite female swimmers. This gender imbalance suggests that the observed performance gains may not be fully generalizable across all demographics, underscoring an urgent need for sex-disaggregated reporting in future research.

### 4.2 Limitations

This study has several limitations. First, the disparity in total training load remains a critical confounding factor. In most trials, the “RMT plus swimming” group received an additional physiological stimulus—estimated to impose a metabolic cost of up to 7% (Okrzymowska et al., 2019; Shei et al., 2016)—compared to “swim-only” controls. This lack of load-matching makes it difficult to determine whether improvements stem from RMT-specific adaptations or simply increased cumulative volume. Future studies should utilize load-matched protocols or active controls to isolate RMT’s independent efficacy. Second, the methodological quality of the included studies constitutes a primary constraint on the strength of our conclusions. Only two trials were identified as having a low risk of bias, while a majority exhibited high risk in critical domains such as randomization processes and deviations from intended interventions. This overall high risk of bias necessitates that even the statistically significant outcomes—particularly those relating to swimming performance—be interpreted with a high degree of caution. Third, the substantial heterogeneity and small sample sizes across several outcomes—most notably aerobic performance—further weaken the strength of the pooled effect sizes. These variations likely stem from a complex interplay of contextual factors, including baseline swimming load (which may exhibit a dose-dependent response), sex-specific respiratory mechanics, and the specific RMT resistance utilized. Given the suboptimal study quality and high heterogeneity, the reported benefits for aerobic capacity and other performance metrics should be downgraded in emphasis and viewed as hypothesis-generating rather than confirmatory. Finally, the inclusion of studies involving para-swimmers introduces specific physiological and biomechanical heterogeneity. While RMT demonstrates potential versatility across diverse athletic populations, the distinct respiratory demands and compensatory mechanisms inherent in para-athletes mean that extrapolating these results to all-elite, able-bodied cohorts must be done with extreme caution.

### 4.3 Implications for practice and research

#### 4.3.1 Practical considerations

From a practical perspective, the statistically significant improvements identified in this meta-analysis provide objective, albeit preliminary, support for the efficacy of RMT in enhancing respiratory muscle strength (MIP) and dynamic ventilatory capacity (FEV1, FIV1) in competitive swimmers. However, given the heterogeneous certainty ratings across performance outcomes, these findings should be viewed as a preliminary evidence-based framework rather than a definitive clinical prescription. Our analysis suggests that RMT-induced adaptations are relatively robust across varying durations and frequencies, offering considerable versatility for integration into aquatic conditioning programs. Current evidence points to an empirical baseline intensity of 50%–80% MIP over 4–6 weeks; yet, these parameters should be treated as observational trends rather than validated optimal dosages. To ensure a balanced training stimulus, coaches should implement RMT as an individualized adjunct, carefully accounting for the additional metabolic cost to avoid unintended cumulative fatigue. Until future randomized trials effectively decouple RMT from total training load, it remains an exploratory but promising strategy for respiratory optimization in elite swimming.

#### 4.3.2 Research implications

i. Future investigations should transition from exploratory studies to well-powered, load-matched randomized controlled trials. It is critical to isolate the independent effects of RMT from the confounding influence of increased total training volume.
ii. Future research should establish standardized criteria for classifying swimmers’ competitive levels (e.g., based on FINA points or annual competition tiers) and conduct stratified analyses to clarify the differential effects of RMT across athletic levels.
iii. There is an urgent need for sex-stratified reporting and age-specific protocols. Given the inherent differences in respiratory mechanics between sexes, future studies must provide disaggregated data to facilitate the development of truly tailored strategies.
iv. Subsequent research should incorporate direct physiological endpoints (e.g., EMG, muscle biopsies, or metabolic reflex markers) to clearly distinguish confirmed adaptations from theoretical interpretations.

## 5 Conclusion

RMT is an effective stimulus for enhancing respiratory muscle strength and dynamic lung function in competitive swimmers across diverse levels. Subgroup analyses identify intervention type as a key moderator, combined RMT appears more effective than IMT alone in promoting ventilatory improvements. However, RMT’s impact on 50–200 m swimming performance remains limited. Potential aerobic benefits must be interpreted with caution due to the substantial heterogeneity across included studies. While this review provides preliminary evidence for integrating RMT into aquatic conditioning, future high-quality, load-matched RCTs are essential to isolate the independent effects of RMT from total training volume.

## Data Availability

The original contributions presented in the study are included in the article/Supplementary Material, further inquiries can be directed to the corresponding authors.
