# Supplementary material for: Effects of respiratory muscle training on swimming performance, respiratory muscle function, and pulmonary function of competitive swimmers: a systematic review and meta-analysis
Source: Front Physiol. 2026 Mar 20;17:1770925. doi: 10.3389/fphys.2026.1770925 (PMC13046513; doi:10.3389/fphys.2026.1770925)

Supplementary Material

Effects of Respiratory Muscle Training on Swimming Performance, Respiratory Muscle Function, and Pulmonary Function of Competitive Swimmers: A Systematic Review and Meta-Analysis

Table S1. Search strategies 2

Table S2. Strength of outcome evidence 4

Table S3. Subgroup characteristics 5

Table S4. Subgroup analyses of potential moderators on respiratory muscle function (MIP and MEP), and pulmonary function (FEV1, FIV1, FVC, and MVV). 8

Figure S1. Results of Cochrane risk of bias tool 11

Figure S2. Funnel plots. 12

Figure S3. Sensitivity analysis results of Respiratory Function 15

Figure S4. Sensitivity analysis results of Swimming Performance 18

Table S1. Search strategies

Database	Step	Search Terms	Records Retrieved
PubMed/MEDLINE	#1	("respiratory muscle training"[All Fields] OR "inspiratory muscle training"[All Fields] OR "expiratory muscle training"[All Fields] OR "RMT"[All Fields] OR "IMT"[All Fields] OR "respiratory muscle conditioning"[All Fields] OR "inspiratory muscle strength training"[All Fields] OR "breathing exercises"[MeSH Terms] OR "respiratory muscles"[MeSH Terms])	4,522
	#2	("swimming"[MeSH Terms] OR "swimmer*"[All Fields] OR "competitive swimming"[All Fields] OR "elite swimmers"[All Fields] OR "aquatic sports"[MeSH Terms] OR "swimming performance"[All Fields])	18,340
	#3	("athletic performance"[MeSH Terms] OR "sports performance"[All Fields] OR "pulmonary function"[All Fields] OR "lung function"[All Fields] OR "maximal inspiratory pressure"[All Fields] OR "PImax"[All Fields] OR "maximal expiratory pressure"[All Fields] OR "PEmax"[All Fields] OR "spirometry"[All Fields] OR "FEV1"[All Fields])	26,415
	#4	#1 AND #2 AND #3	218
Web of Science (Core Collection)	#1	TS=("respiratory muscle training" OR "inspiratory muscle training" OR "expiratory muscle training" OR "RMT" OR "IMT" OR "breathing exercise*" OR "respiratory muscle strength training" OR "inspiratory conditioning")	5,118
	#2	TS=("swimmer*" OR "swimming" OR "competitive swimming" OR "elite swimming" OR "swimming race")	22,410
	#3	TS=("swimming performance" OR "athletic performance" OR "pulmonary function" OR "lung function" OR "maximal inspiratory pressure" OR "PImax" OR "peak flow")	31,502
	#4	#1 AND #2 AND #3	325
Cochrane Library	#1	(["Respiratory Muscle Training" OR "Inspiratory Muscle Training" OR "Expiratory Muscle Training" OR "RMT" OR "IMT" OR "Respiratory Muscle Strength Training" OR "Breathing Exercise*"]:ti,ab,kw) OR (MeSH descriptor: [Respiratory Muscles] explode all trees) OR (MeSH descriptor: [Breathing Exercises] explode all trees)	850
	#2	(["swimming" OR "swimmer*" OR "competitive swimming" OR "aquatic sports"]:ti,ab,kw) OR (MeSH descriptor: [Swimming] explode all trees)	2,120
	#3	#1 AND #2	82
SPORTDiscus	#1	(TI "respiratory muscle training" OR AB "respiratory muscle training" OR TI "inspiratory muscle training" OR AB "inspiratory muscle training" OR "RMT" OR "IMT")	1,240
	#2	(TI "swimming" OR AB "swimming" OR TI "swimmer*" OR AB	15,600

		"swimmer*" OR "competitive swimming")	
	#3	S1 AND S2	195
Embase	#1	('respiratory muscle training'/exp OR 'breathing exercise'/exp OR 'respiratory muscle strength training' OR 'inspiratory muscle training' OR 'expiratory muscle training' OR 'rmt' OR 'imt' OR 'emt' OR 'inspiratory threshold loading' OR 'respiratory muscle conditioning')	6,800
	#2	('swimming'/exp OR 'swimmer'/exp OR 'swimming' OR 'swimmer*' OR 'competitive swimming' OR 'elite swimmer' OR 'aquatic sports'/exp)	28,400
	#3	S1 AND S2	264
Scopus	#1	TITLE-ABS-KEY ("respiratory muscle training" OR "inspiratory muscle training" OR "expiratory muscle training" OR "RMT" OR "IMT" OR "breathing exercise*" OR "respiratory muscle strength*" OR "respiratory conditioning")	9,500
	#2	TITLE-ABS-KEY ("swimming" OR "swimmer*" OR "competitive swimming" OR "elite swimming" OR "aquatic sport*")	35,000
	#3	S1 AND S2	166
Other sources	(ScienceDirect, ProQuest, etc.)		100
Total	Combined records from all databases		1,350
Final	Records after duplicates removed		1,053

Table S2. Strength of outcome evidence

Quality assessment								No of participants		Effect	Quality	Importance
No of experiments	Design	Risk of bias	Inconsistency	Indirectness	Imprecision	Publication Bias	Other considerations	EXP	CON	Absolute effect (95% CI)		
	MIP											
12	Randomised trials	Serious[1]	Serious[2]	No serious	No serious	No serious	None	192	175	0.65 (0.29 to 1.01)	⊕⊕⊖⊖ Low	Critical
	MEP											
8	Randomised trials	Serious[1]	No serious	No serious	Serious[3]	No serious	None	94	83	0.16 (-0.14 to 0.46)	⊕⊕⊖⊖ Low	Critical
	FEV1											
9	Randomised trials	Serious[1]	No serious	No serious	No serious	No serious	None	126	115	0.45 (0.19 to 0.72)	⊕⊕⊕⊖ Moderate	Critical
	FIV1											
3	Randomised trials	No serious	No serious	No serious	No serious	Serious[3]	None	52	52	0.41 (0.04 to 0.79)	⊕⊕⊕⊖ Moderate	Critical
	FVC											
12	Randomised trials	Serious[1]	No serious	No serious	Serious[3]	No serious	None	112	108	0.26 (-0.001 to 0.51)	⊕⊕⊖⊖ Low	Important
	MVV											
10		Serious[1]	Serious[2]	No serious	Serious[3]	No serious	None	91	88		⊕⊖⊖⊖	Important

	Randomised trials									-0.23 (-1.45 to 0.98)	Very Low	
	50 m Freestyle Time											
4	Randomised trials	Serious[1]	No serious	No serious	Serious[3]	No serious	None	40	36	-0.38 (-0.98 to 0.21)	⊕⊕⊖⊖ Low	Important
	100 m Freestyle Time											
5	Randomised trials	Serious[1]	No serious	No serious	Serious[3]	No serious	None	50	45	-0.17 (-0.54 to 0.19)	⊕⊕⊖⊖ Low	Important
	200 m Freestyle Time											
5	Randomised trials	Serious[1]	No serious	No serious	Serious[3]	No serious	None	50	45	-0.07 (-0.57 to 0.44)	⊕⊕⊖⊖ Low	Important
	Aerobic Performance											
3	Randomised trials	No serious	Serious[2]	No serious	Serious[3]	No serious	None	30	27	1.02 (0.03 to 2.00)	⊕⊕⊖⊖ Low	Important

Note: EXP: Experimental group; CON: Control group; SMD, standardized mean difference; CI, Confidence interval, MIP: Maximal inspiratory pressure; MEP: Maximal expiratory pressure; FEV1: Forced expiratory volume in 1 second; FIV1: Forced Inspiratory Volume in the 1st second; FVC: Forced Vital Capacity; MVV: Maximal Voluntary Ventilation;

1 High risk of detection bias.

2 Significant heterogeneity.

3 Small sample size and wide CI crosses null.

Table S3. Subgroup characteristics

**Study**	**Athletic levels**	**Intervention method**	**Duration (week)**	**Frequency**	**Training intensity**

				(session/week)	
Ohya et al., 2022(33) HIGH	High level	IMT	≤ 6w	≥ 10	75% MIP
Ohya et al., 2022(33) MOD	High level	IMT	≤ 6w	≥ 10	50% MIP
Yáñez-Sepúlveda et al., 2021(38)	High level	IMT	≤ 6w	< 10	50% MIP，increased by 5% weekly
Ando et al., 2020(28)	Elite level	IMT	≤ 6w	≥ 10	50% MIP
Cunha et al., 2019(14)	Elite level	IMT	> 6w	< 10	50% MIP
Lomax et al., 2019(31) LOW	High level	IMT	≤ 6w	≥ 10	50% MIP, increased periodically
Lomax et al., 2019(31) HIGH	High level	IMT	≤ 6w	≥ 10	50% MIP, increased periodically
Okrzymowska et al., 2019(34)	Elite level	IMT	> 6w	≥ 10	30~60% MIP
Vašíčkováet al., 2017(36)	High level	RMT (RMST & RMET)	≤ 6w	< 10	30% of MIP and MEP, increased by 2 cm H_2O weekly
Shei et al., 2016(35)	High level	IMT	> 6w	< 10	80% MIP, progressively increased work-rest ratios until task failure
Kapus et al., 2013(29)	High level	IMT	≤ 6w	≥ 10	50% MIP, increased weekly
Lemaitre et al., 2013(30)	High level	RMT (RMET)	> 6w	< 10	60% MVV, adjusted weekly
Kilding et al., 2010(13)	High level	IMT	≤ 6w	≥ 10	50% MIP, increased weekly

Mickleborough et al., 2008(32)	Elite level	IMT	> 6w	< 10	80% SMIP
Wylegala et al., 2007(37) RRMT	High level	RMT (RMST)	≤ 6w	< 10	50 cm H_2O
Wylegala et al., 2007(37) ERMT	High level	RMT (EMET)	≤ 6w	< 10	60% MVV, adjusted weekly
Wells et al.,2005(5) 6weeks—F	High level	RMT (IMT& EMT)	≤ 6w	≥ 10	50~60% MIP/MEP
Wells et al.,2005(5) 12weeks—F	High level	RMT	> 6w	≥ 10	70~80% MIP/MEP
Wells et al.,2005(5) 6weeks—M	High level	RMT	≤ 6w	≥ 10	50~60% MIP/MEP
Wells et al.,2005(5) 12weeks—M	High level	RMT	> 6w	≥ 10	70~80% MIP/MEP

Table S4. Subgroup analyses of potential moderators on respiratory muscle function (MIP and MEP), and pulmonary function (FEV1, FIV1, FVC, and MVV).

**Subgroup**	**Moderator**		**No. of studies**	**SMDs (95% CI)**	I^2	p_{group}	P_{diff}
MIP	Athletic levels	Elite swimmers	4	0.91 (0.03 to 1.80)	70.47	0.01	0.49
		High-level	15	0.57 (0.17 to 0.97)	56.53	0.02	
	Intervention method	IMT	12	0.95 (0.38 to 1.52)	68.73	0.001	0.07
		RMT	7	0.28 (-0.16 to 0.69)	21.92	0.19	
	Duration	≤ 6w	13	0.56 (0.18 to 0.93)	44.44	0.03	0.49
		> 6w	6	0.95 (-0.08 to 1.98)	84.26	0.19	
	Frequency	< 10	7	0.92 (0.15 to 1.68)	76.15	0.02	0.38
		≥ 10	12	0.55 (0.10 to 0.99)	53.07	0.02	
MEP	Athletic levels	Elite swimmers	2	-0.01(-0.67 to 0.66)	0.00	0.53	0.59
		High-level	8	0.20 (-0.14 to 0.54)	0.00	0.46	
	Intervention method	IMT	3	-0.11(-0.68 to 0.47)	0.00	0.69	0.29
		RMT	7	0.26(-0.09 to 0.61)	0.00	0.48	
	Duration	≤ 6w	5	0.12(-0.30 to 0.55)	0.00	0.86	0.81
		> 6w	5	0.21(-0.32 to 0.73)	33.69	0.20	

	Frequency	< 10	4	0.35(-0.23 to 0.93)	39.59	0.17	0.36
		≥ 10	6	0.02(-0.39 to 0.42)	0.00	0.64	
FEV1	Athletic levels	Elite swimmers	3	0.23(-0.27 to 0.73)	0.00	0.68	0.30
		High-level	10	0.54(0.23 to 0.86)	0.00	0.03	
	Intervention method	IMT	7	0.19(-0.17 to 0.55)	0.00	0.98	**0.03**
		RMT	6	0.78(0.38 to 1.17)	0.00	0.02	
	Duration	≤ 6w	7	0.45(0.08 to 0.82)	0.00	0.03	0.90
		> 6w	6	0.48(0.04 to 0.93)	25.6	0.02	
	Frequency	< 10	6	0.27(-0.10 to 0.63)	0.00	0.91	0.14
		≥ 10	7	0.67(0.28 to 1.06)	0.00	0.03	
FIV1	Athletic levels	Elite swimmers	1	-0.02(-0.89 to 0.86)	——	——	——
		High-level	5	0.54(0.10 to 0.99)	25.33	0.02	
	Intervention method	IMT	2	0.52(-0.16 to 1.21)	72.89	0.06	0.74
		RMT	4	0.38(-0.10 to 0.87)	24.23	0.42	
	Duration	≤ 6w	2	0.30(-0.38 to 0.98)	11.24	0.29	0.64
		> 6w	4	0.50(0.01 to 0.98)	24.23	0.04	

	Frequency	< 10	2	0.52(-0.16 to 1.21)	72.89	0.06	0.74
		≥ 10	4	0.38(-0.10 to 0.87)	0.00	0.42	
FVC	Athletic levels	Elite swimmers	3	0.20(-0.30 to 0.69)	0.00	0.64	0.85
		High-level	9	0.25(-0.06 to 0.57)	0.00	0.23	
	Intervention method	IMT	6	0.12(-0.25 to 0.50)	0.00	0.34	0.39
		RMT	6	0.36(-0.03 to 0.74)	0.00	0.07	
	Duration	≤ 6w	6	0.24(-0.15 to 0.62)	0.00	0.12	0.99
		> 6w	6	0.24(-0.13 to 0.61)	0.00	0.16	
	Frequency	< 10	6	0.21(-0.15 to 0.57)	0.00	0.24	0.84
		≥ 10	6	0.27(-0.31 to 0.67)	0.00	0.21	
MVV	Intervention method	IMT	4	0.53(-0.45 to 1.50)	73.11	0.23	0.23
		RMT	6	-0.96(-3.17 to 1.24)	92.83	0.32	
	Duration	≤ 6w	7	-0.78(-2.56 to 1.00)	95.32	0.54	0.18
		> 6w	3	0.77(-0.59 to 2.12)	78.94	0.36	
	Frequency	< 10	4	-1.08(-5.24 to 3.08)	97.69	0.45	0.60
		≥ 10	6	0.03(-0.35 to 0.40)	0.00	0.14	

Figure S1. Results of Cochrane risk of bias tool

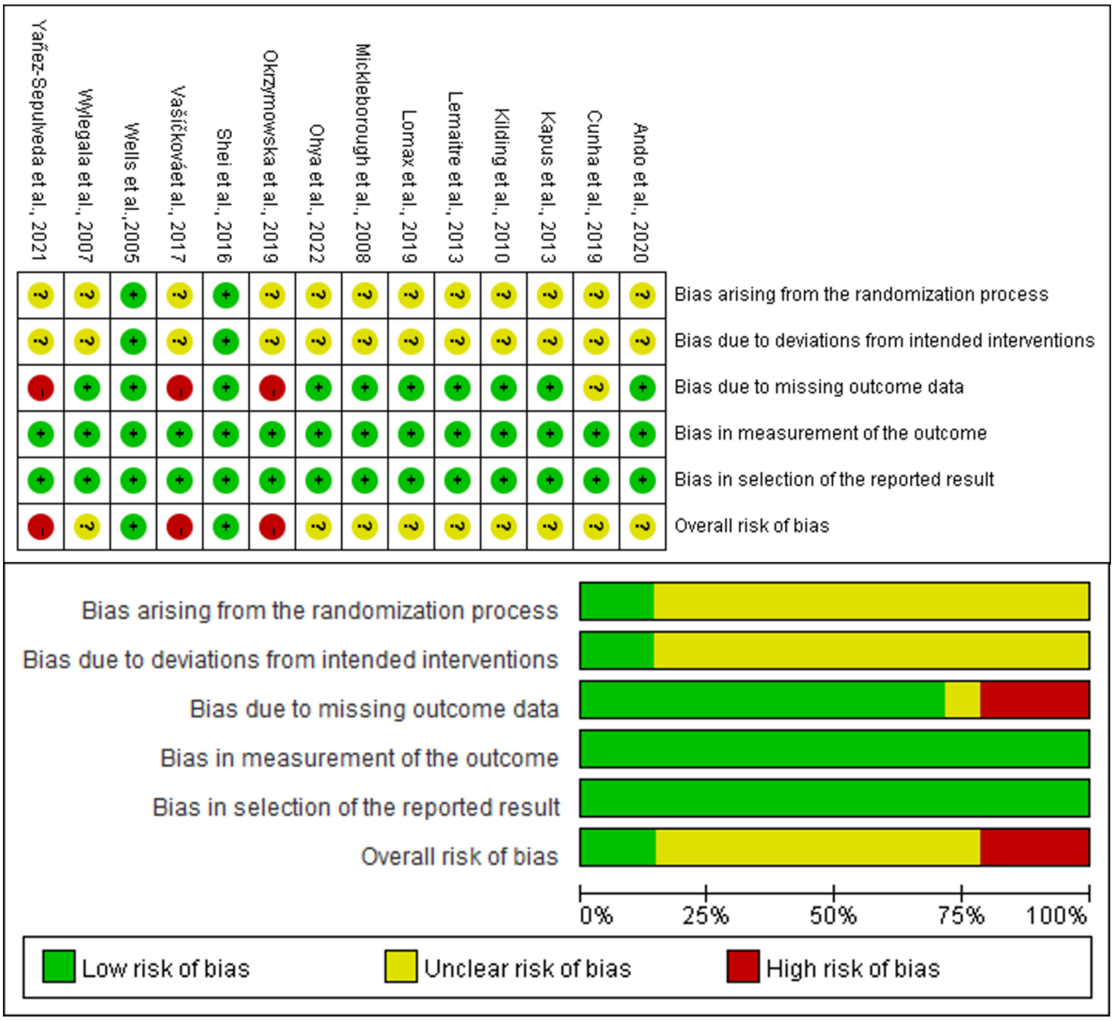

Figure S2. Funnel plots.

(1) MIP

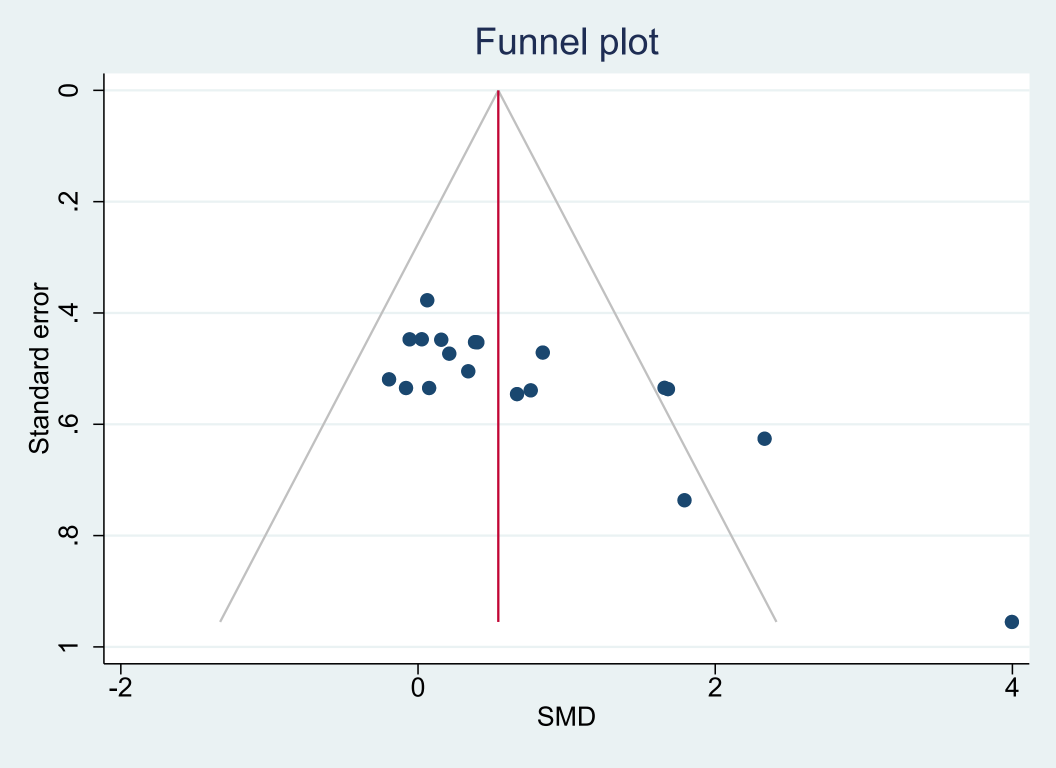

(2) MEP

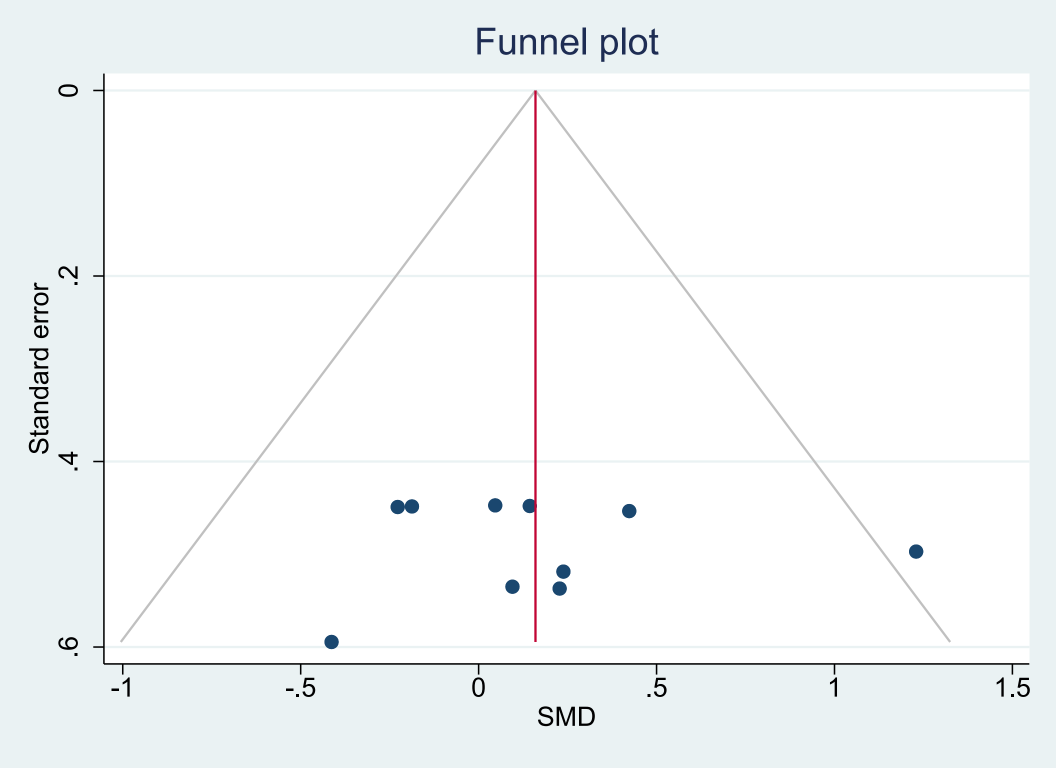

(3) FEV1

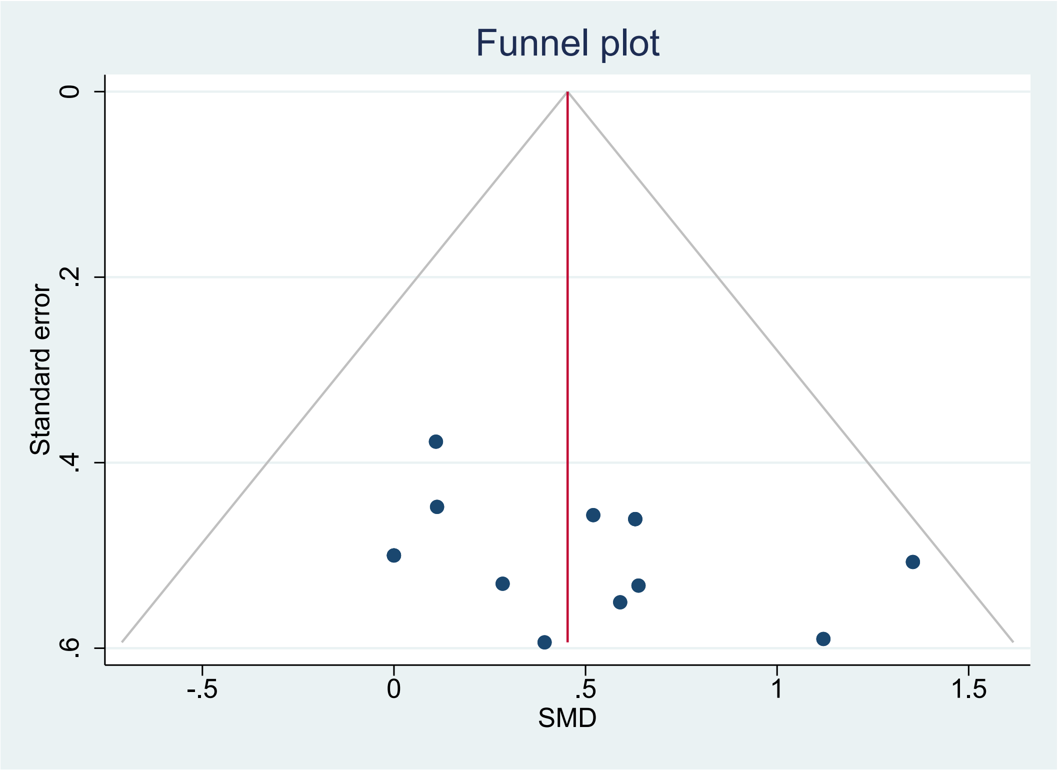
Funnel plot
0
.2
.4
.6
Standard error
-.5
0
.5
1
1.5
SMD

(4) FIV1

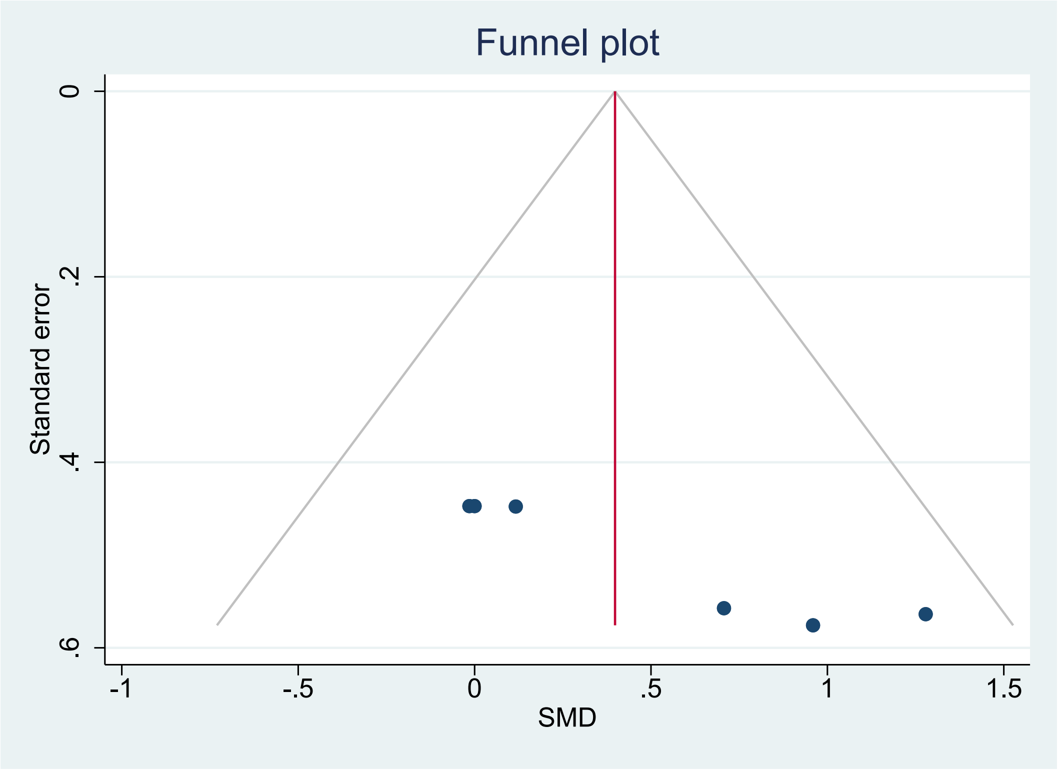
Funnel plot
0
.2
.4
.6
Standard error
-1
-.5
0
.5
1
1.5
SMD

(5) FVC

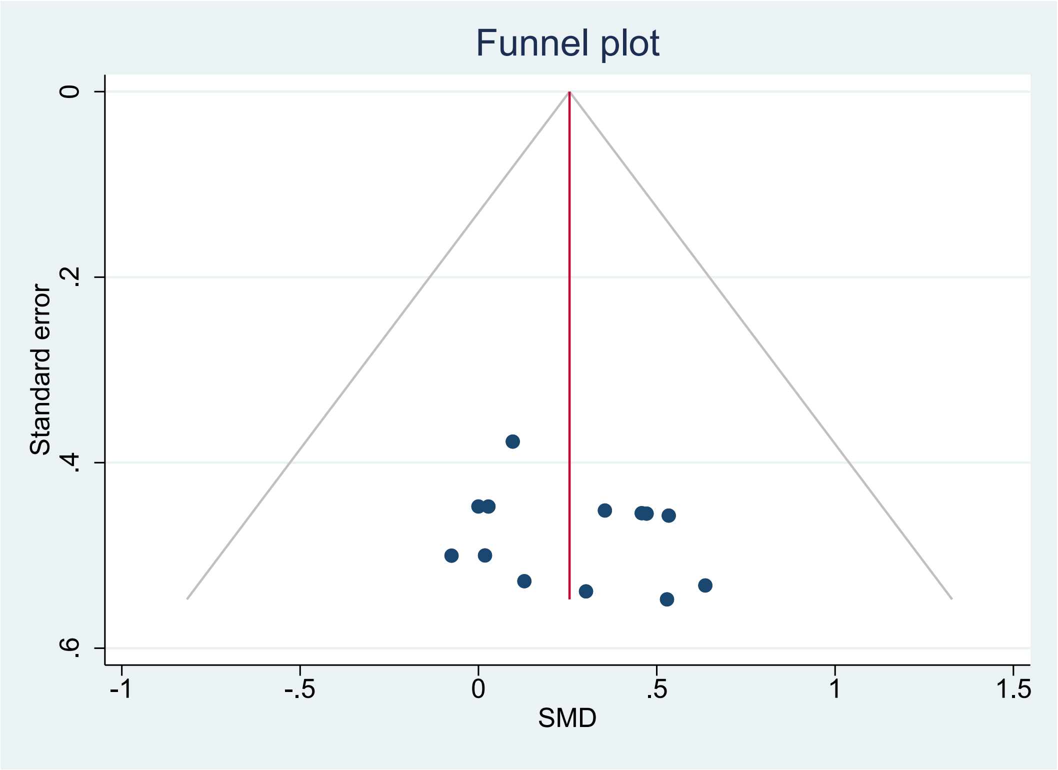

Funnel plot
Standard error
0
.2
.4
.6
-1
-.5
0
.5
1
1.5
SMD

(6) MVV

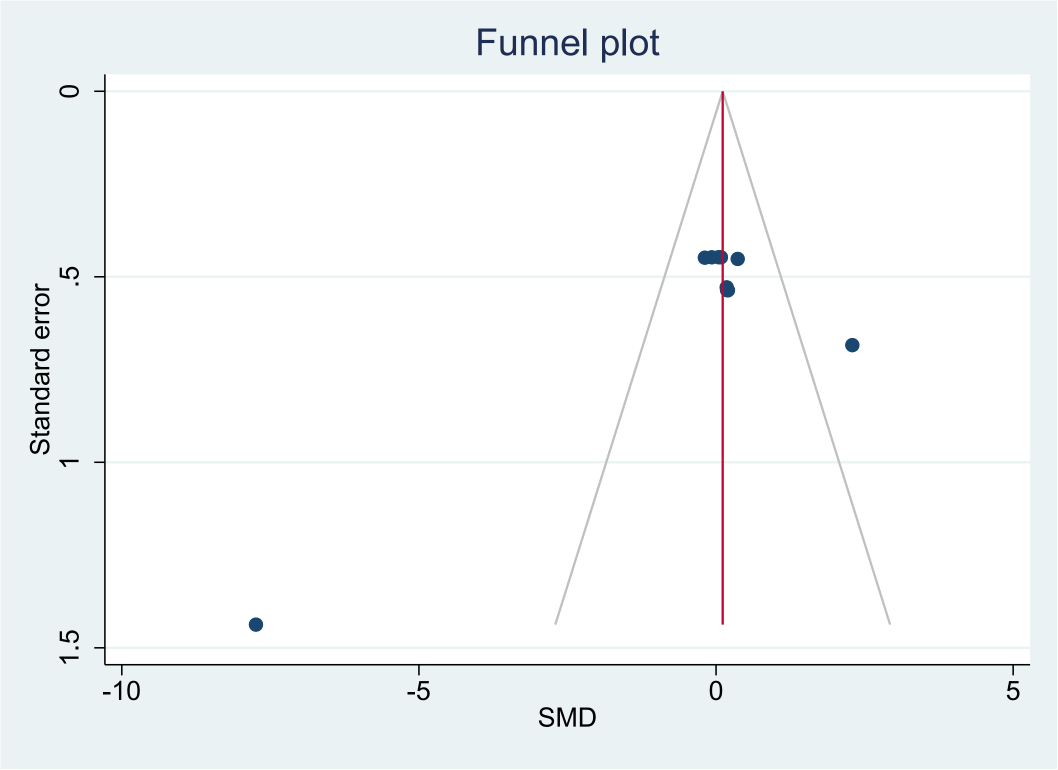

Funnel plot
Standard error
0
.5
1
1.5
-10
-5
0
5
SMD

Figure S3. Sensitivity analysis results of Respiratory Function

(1) MIP

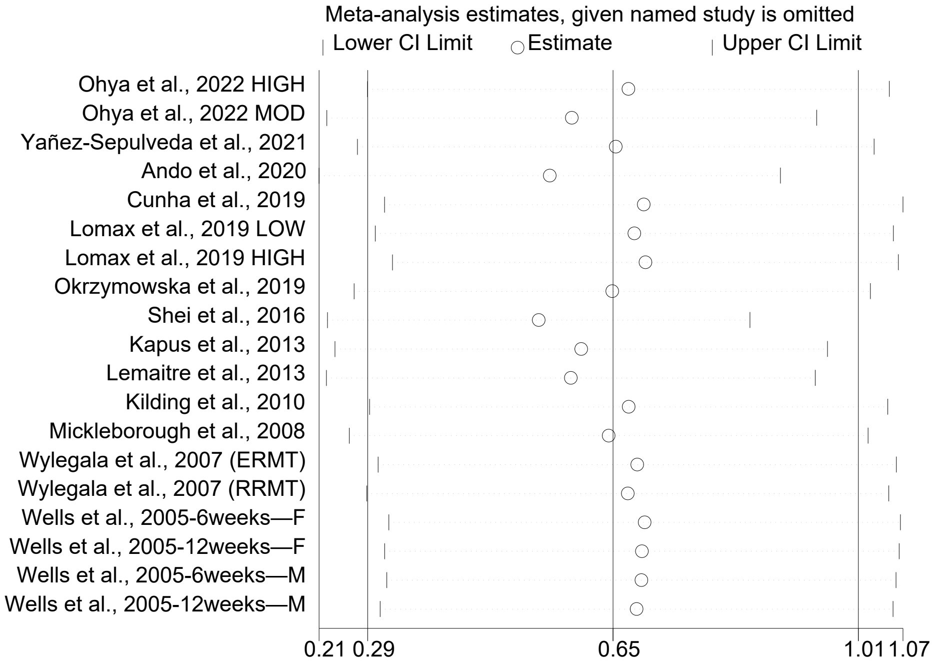

(2) MEP

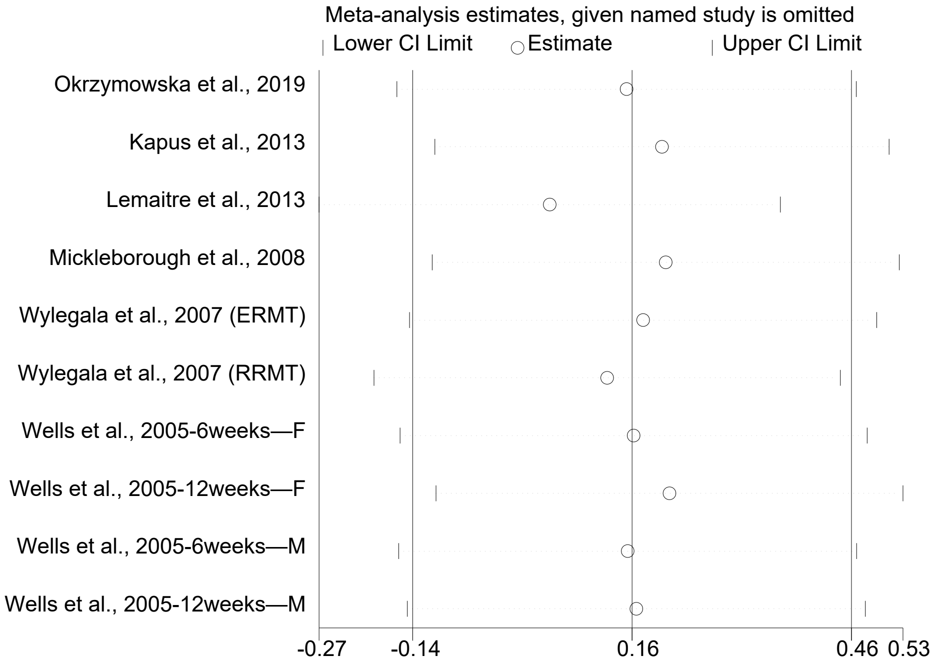

(3) FEV1

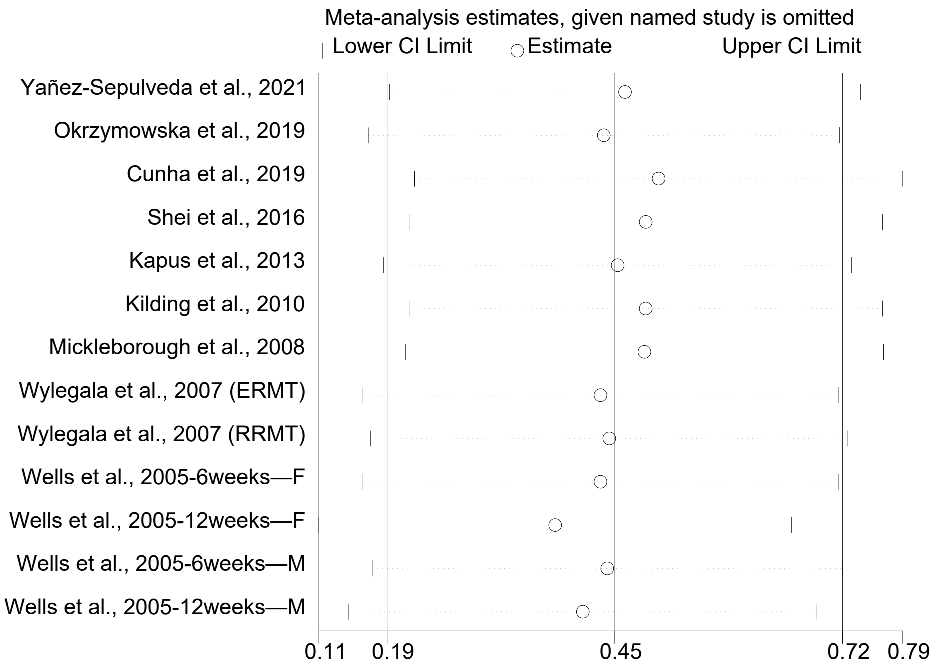
Meta-analysis estimates, given named study is omitted
Lower CI Limit
Estimate
Upper CI Limit
Yañez-Sepulveda et al., 2021
Okrzymowska et al., 2019
Cunha et al., 2019
Shei et al., 2016
Kapus et al., 2013
Kilding et al., 2010
Mickleborough et al., 2008
Wylegala et al., 2007 (ERMT)
Wylegala et al., 2007 (RRMT)
Wells et al., 2005-6weeks—F
Wells et al., 2005-12weeks—F
Wells et al., 2005-6weeks—M
Wells et al., 2005-12weeks—M
0.11
0.19
0.45
0.72
0.79

(4) FIV1

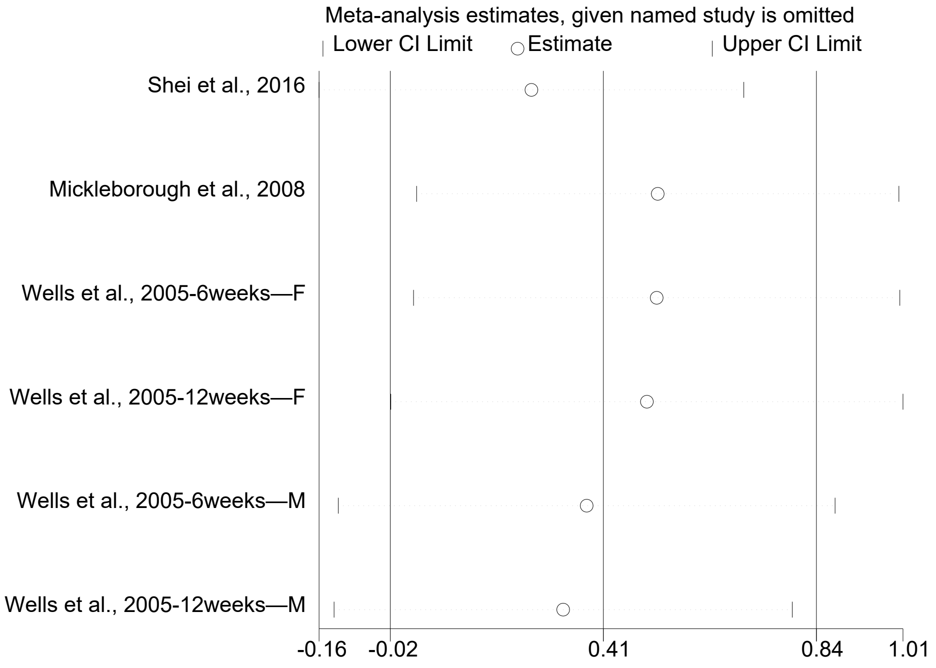
Meta-analysis estimates, given named study is omitted
Lower CI Limit
Estimate
Upper CI Limit
Shei et al., 2016
Mickleborough et al., 2008
Wells et al., 2005-6weeks—F
Wells et al., 2005-12weeks—F
Wells et al., 2005-6weeks—M
Wells et al., 2005-12weeks—M
-0.16
-0.02
0.41
0.84
1.01

(5) FVC

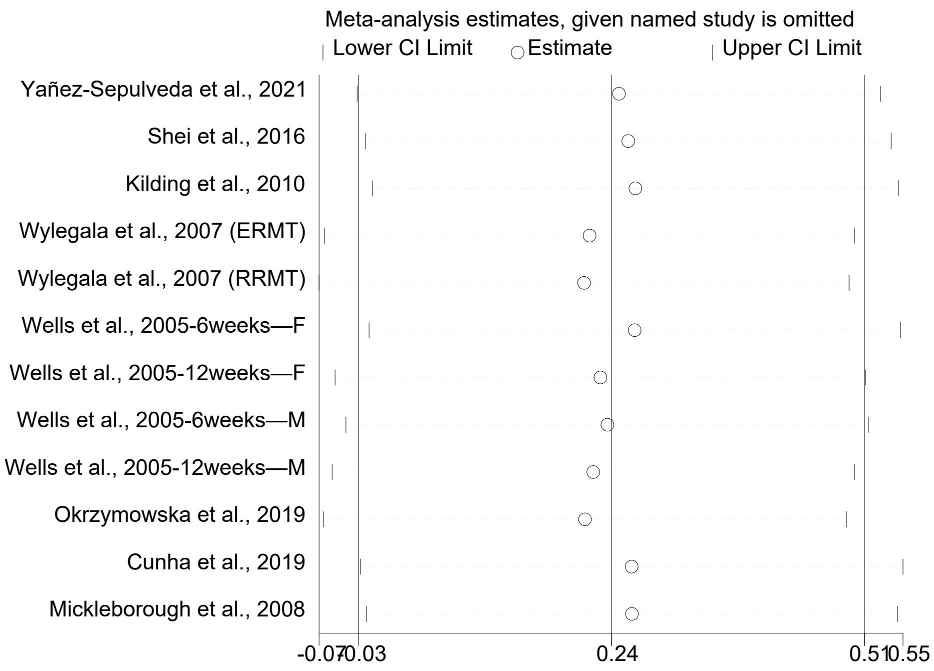
Meta-analysis estimates, given named study is omitted
Lower CI Limit
Estimate
Upper CI Limit
Yañez-Sepulveda et al., 2021
Shei et al., 2016
Kilding et al., 2010
Wylegala et al., 2007 (ERMT)
Wylegala et al., 2007 (RRMT)
Wells et al., 2005-6weeks—F
Wells et al., 2005-12weeks—F
Wells et al., 2005-6weeks—M
Wells et al., 2005-12weeks—M
Okrzymowska et al., 2019
Cunha et al., 2019
Mickleborough et al., 2008
-0.07
0.03
0.24
0.51
0.55

(6) MVV

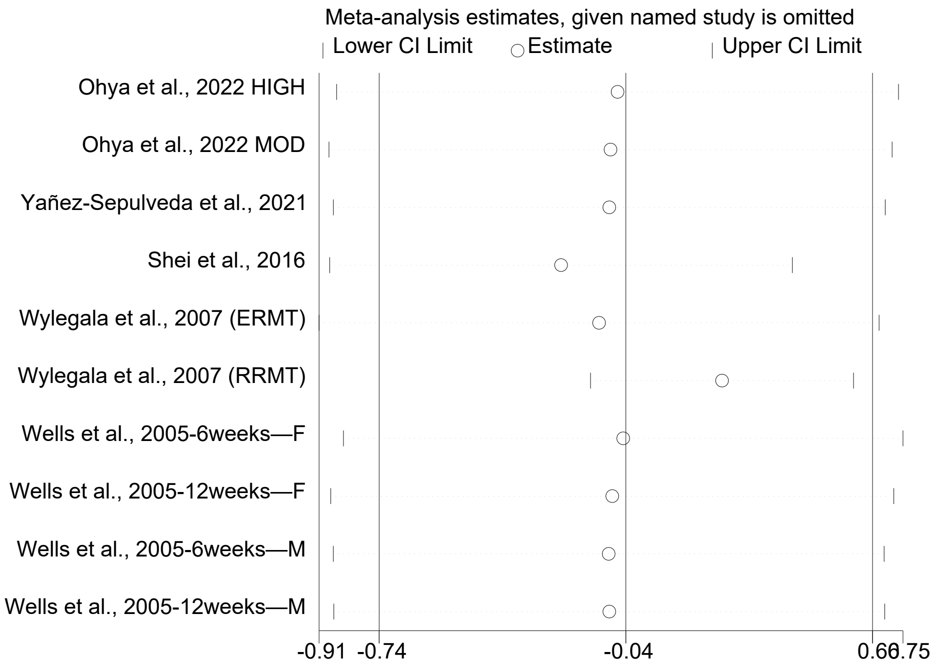
Meta-analysis estimates, given named study is omitted
Lower CI Limit
Estimate
Upper CI Limit
Ohya et al., 2022 HIGH
Ohya et al., 2022 MOD
Yañez-Sepulveda et al., 2021
Shei et al., 2016
Wylegala et al., 2007 (ERMT)
Wylegala et al., 2007 (RRMT)
Wells et al., 2005-6weeks—F
Wells et al., 2005-12weeks—F
Wells et al., 2005-6weeks—M
Wells et al., 2005-12weeks—M
-0.91
-0.74
-0.04
0.66
0.75

Figure S4. Sensitivity analysis results of Swimming Performance

(1) 100 m freestyle time

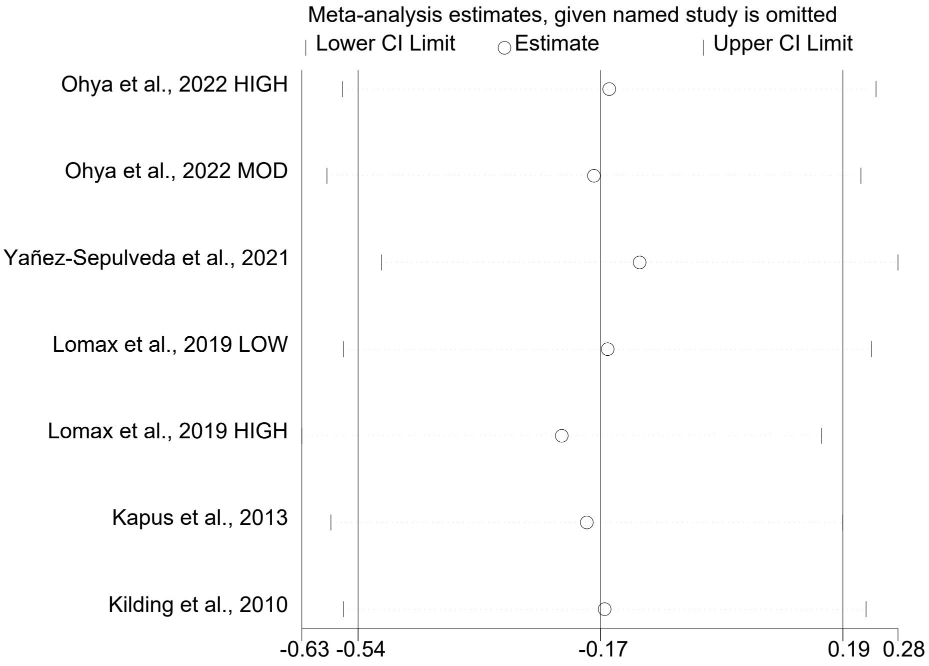

(2) 200 m freestyle time

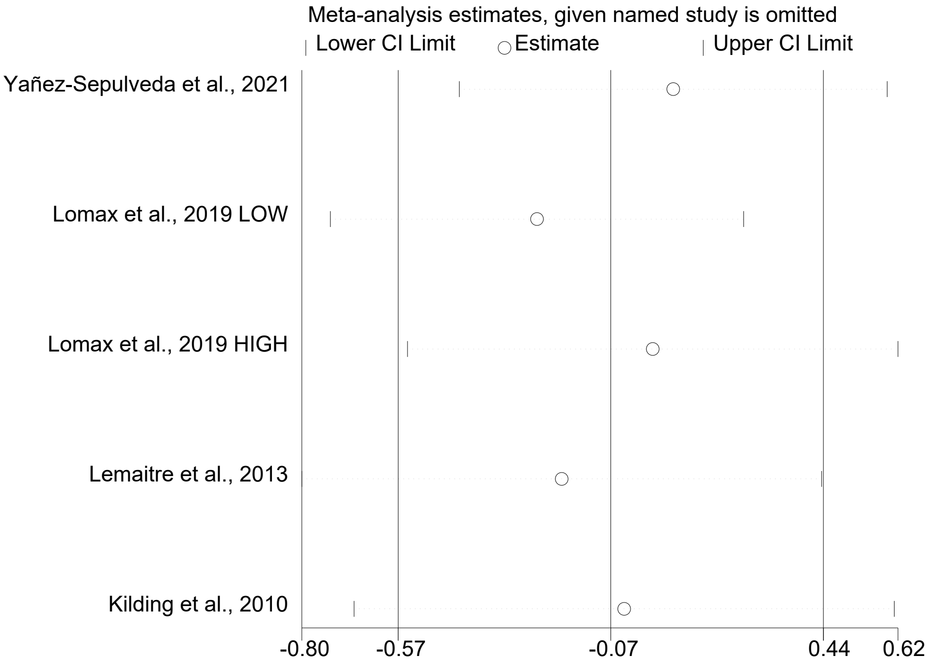

Supplement: Supplementary file 1 [file DataSheet1.pdf]
